# An integrative systematic revision and biogeography of *Rhynchocalamus* snakes (Reptilia, Colubridae) with a description of a new species from Israel

**DOI:** 10.7717/peerj.2769

**Published:** 2016-12-22

**Authors:** Karin Tamar, Jiří Šmíd, Bayram Göçmen, Shai Meiri, Salvador Carranza

**Affiliations:** 1 The Steinhardt Museum of Natural History, Israel National Center for Biodiversity Studies, Tel Aviv University, Tel Aviv, Israel; 2 Department of Zoology, Tel Aviv University, Tel Aviv, Israel; 3 South African National Biodiversity Institute, Cape Town, South Africa; 4 Department of Biology, Zoology Section, Ege University, Bornova-Izmir, Turkey; 5 Animal Biodiversity and Evolution, Institute of Evolutionary Biology (CSIC-Pompeu Fabra University), Barcelona, Spain

**Keywords:** Diversification, Arabia, Evolution, Taxonomy, Phylogeny, Phylogeography, Middle east, Reptiles

## Abstract

**Background:**

The colubrid snakes of the genus *Rhynchocalamus* are seldom studied and knowledge of their ecology and life history is scarce. Three species of *Rhynchocalamus* are currently recognized, *R. satunini* (from Turkey eastwards to Iran), *R. arabicus* (Yemen and Oman), and *R. melanocephalus* (from the Sinai Peninsula northwards to Turkey). All are slender, secretive, mainly nocturnal and rare fossorial snakes. This comprehensive study is the first to sample all known *Rhynchocalamus* species in order to review the intra-generic phylogenetic relationships and historical biogeography of the genus.

**Methods:**

We revised the systematics of *Rhynchocalamus* using an integrative approach and evaluated its phylogeography. The phylogenetic position within the Colubridae and the phylogenetic relationships within the genus were inferred using 29 individuals belonging to the three known species, with additional sampling of two other closely-related genera, *Muhtarophis* and *Lytorhynchus*. We analysed three mitochondrial (*12S*, *16S, cytb*) and one nuclear (*c-mos*) gene fragments. Phylogenetic trees were reconstructed using maximum likelihood and Bayesian inference methods; the latter method also used to provide the first time-calibrated molecular phylogeny of the genus. We generated a nuclear network and carried out a topology test and species delimitation analysis. Morphological comparisons were used to differentiate among species and to describe a new species from Israel. The studied material was comprised of 108 alcohol-preserved specimens, 15 photographs, and data from the literature for the examination of 17 mensural, 14 meristic, and two categorical characters.

**Results:**

The molecular results support *Rhynchocalamus* as monophyletic, and as having split from its sister genus *Lytorhynchus* during the Late Oligocene. The three recognized species of *Rhynchocalamus* comprise four independently evolving groups. The molecular results reveal that the genus began to diverge during the Middle Miocene. We revealed that the best-studied species, *R. melanocephalus,* is paraphyletic. A population, formally ascribed to this species, from the Negev Mountain area in southern Israel is phylogenetically closer to *R. arabicus* from Oman than to the northern populations of the species from Israel, Syria and Turkey. Herein we describe this population as a new species: *Rhynchocalamus dayanae* **sp. nov.**

**Discussion:**

We identify four species within *Rhynchocalamus*: *R. satunini, R. arabicus, R. melanocephalus*, and *R. dayanae* **sp. nov.**, the latter, to the best of our knowledge, is endemic to southern Israel. The onset of *Rhynchocalamus* diversification is very old and estimated to have occurred during the Middle Miocene, possibly originating in the Levant region. Radiation probably resulted from vicariance and dispersal events caused by continuous geological instability, sea-level fluctuations and climatic changes within the Levant region.

## Introduction

Taxonomy today often relies on molecular data for further support and information. Such data are usually preferred over morphology for the reconstruction of evolutionary relationships among organisms (Hebert et al., 2003; Tautz et al., 2003; Blaxter, 2004; Vogler & Monaghan, 2007; Padial et al., 2010). The increasing use and availability of molecular data has led to the development of new methods to study systematics (Sites & Marshall, 2003), and has proven to be an invaluable tool for evaluating the evolutionary relationships between both closely and distantly related species. Recent studies of Middle Eastern snakes have used molecular data to elucidate the inter- and intra-specific relationships among taxa, revealing high levels of genetic differentiation and cryptic diversity that do not accord with the current taxonomy. Such studies have also provided insights into the historical biogeography of the taxa and the processes that triggered their diversification (e.g., Lenk et al., 2001; Utiger et al., 2002; Nagy et al., 2003; Nagy et al., 2004; Pook et al., 2009; Stümpel & Joger, 2009; Kornilios et al., 2013). However, the biodiversity of snakes in the Middle East remains unclear, as systematic and biogeographic data for several genera are still lacking. One such example is that of the colubrid genus *Rhynchocalamus* Günther, 1864.

**Figure 1:**
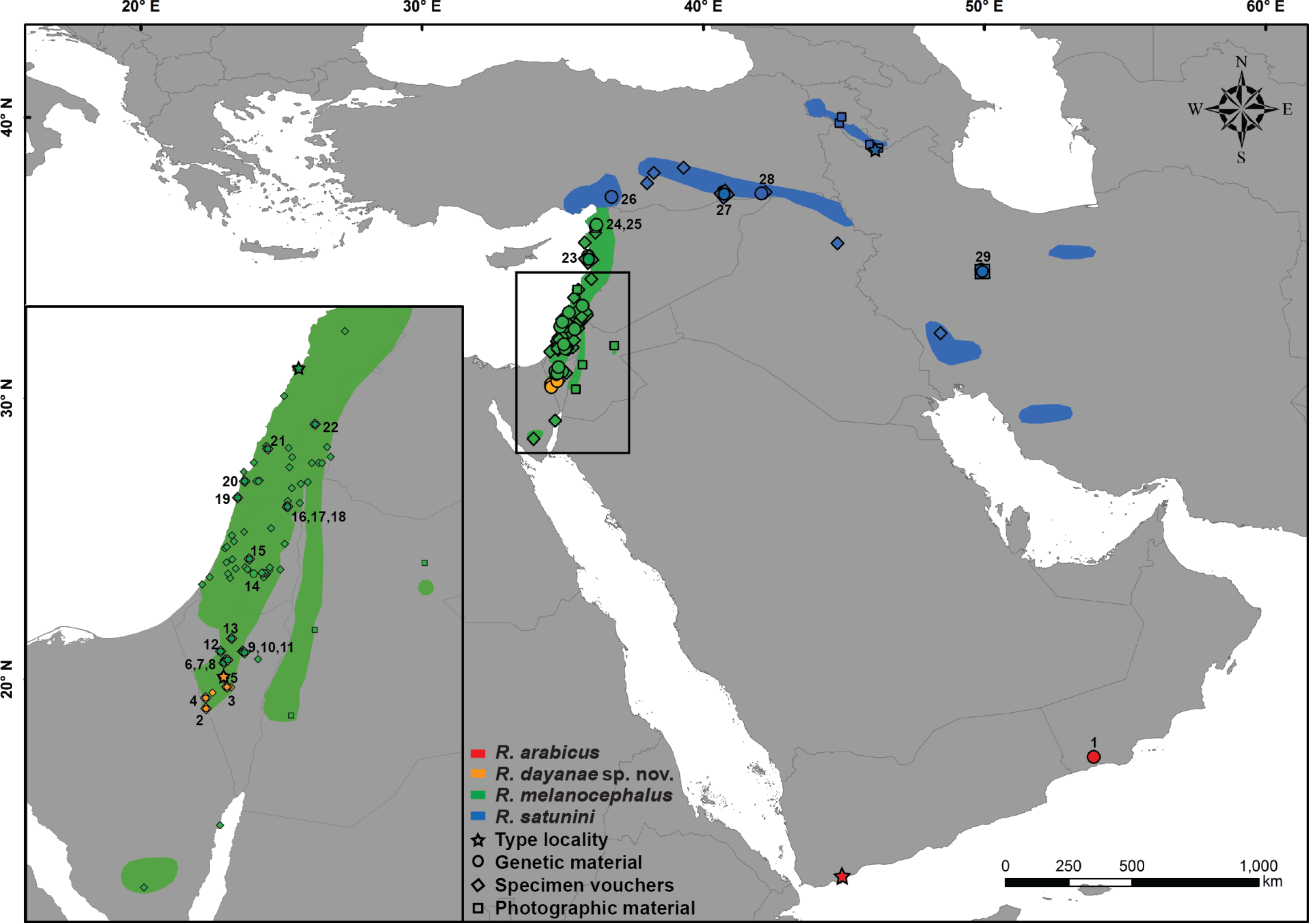
Distribution map of *Rhynchocalamus*. Localities of the material analysed in this study, including type localities (star), samples used for the genetic analyses (circle), specimen vouchers for the morphological examinations (diamond) and photographic material (square). Numbers correspond to samples listed in Table S1 and colours to specimens in Fig. 2 and Figs. S1–S4. Taxon names correspond to changes proposed in this paper. Spatial data modified from various sources (IUCN-http://www.iucnredlist.org; Bar & Haimovitch, 2013; Sindaco, Venchi & Grieco, 2013).

*Rhynchocalamus* snakes are secretive, non-venomous and occasionally found near human habitations. They are poorly known and information regarding their natural history is scarce. These are small-sized, slender, fossorial aglyphous snakes that are mostly nocturnal but can also be found active during the day (Gasperetti, 1988; Disi et al., 2001; Baha El Din, 1994; Baha El Din, 2006; Avci et al., 2007; Avci et al., 2008). Morphologically they are characterized by a thin cylindrical body and short tail, small head indistinct from the neck, an enlarged rostral shield wedged between the internasals scales, and divided anal plate and subcaudal scales (Gasperetti, 1988; Disi et al., 2001; Baha El Din, 2006). *Rhynchocalamus* snakes prefer humid areas with little vegetation and are found in mountainous areas in both Mediterranean and Irano-Turanian ecozones (Fig. 1), on heavy soils but not on sand. In southern Israel, Jordan, and Egypt (i.e., the Sinai Peninsula), they are also known from arid and stony steppes, sparsely vegetated rocky slopes and wadis (Disi et al., 2001; Baha El Din, 2006; Amr & Disi, 2011; Bar & Haimovitch, 2013; Werner, 2016).

**Figure 2:**
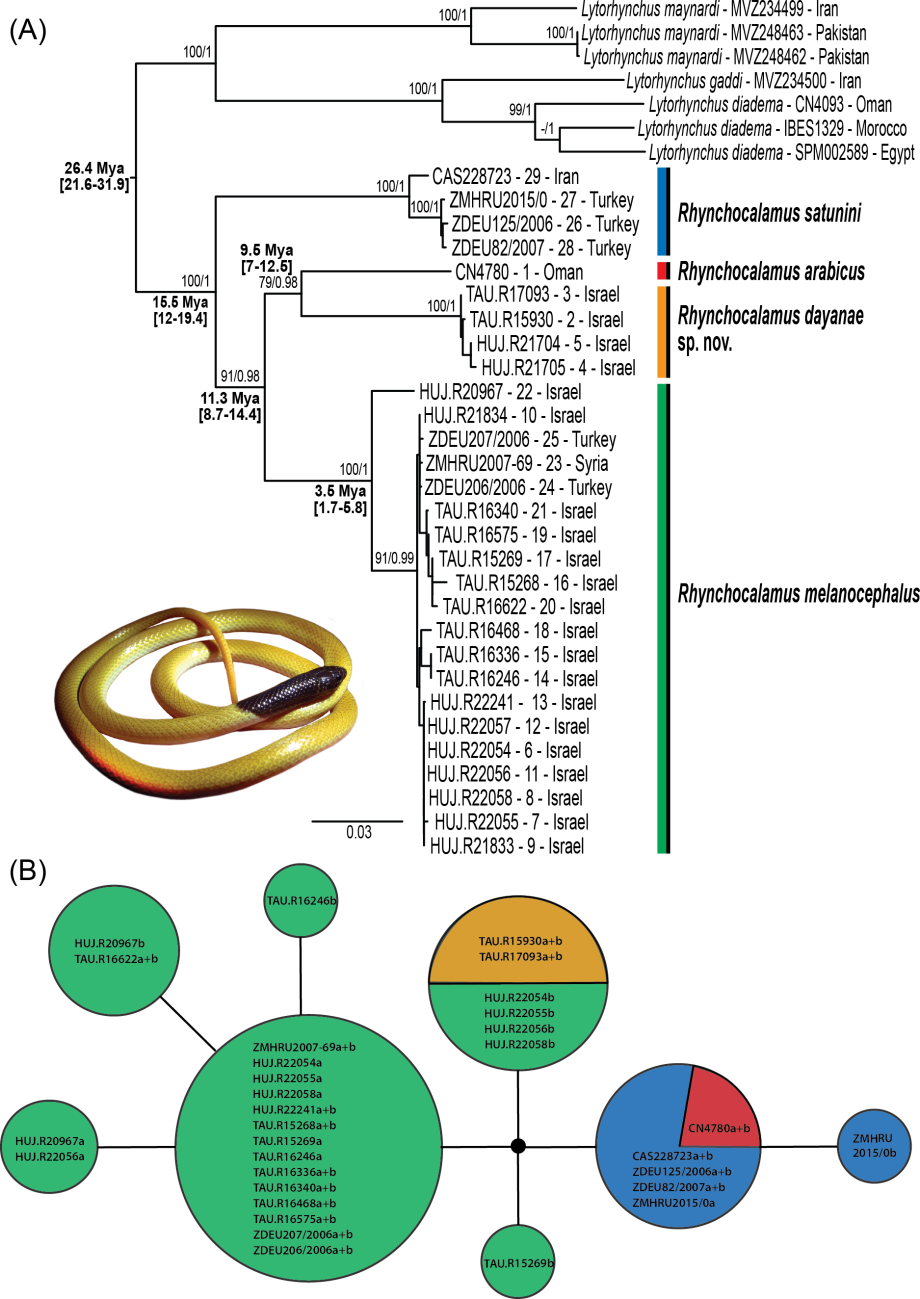
Phylogenetic relationships within *Rhynchocalamus.* (A) Maximum likelihood gene tree inferred from the concatenated dataset of the mitochondrial (*12S, 16S, cytb*) and nuclear (*c-mos*) gene fragments (dataset 3). Support values near the nodes indicate bootstrap and posterior probability (values ≥ 70%/ ≥ 0.95, respectively). Age estimates based on external calibration points of *Hemorrhois* and *Hierophis* subgroups (see ‘Materials and Methods’) are indicated near the relevant nodes and include the mean and, between parentheses, the 95% highest posterior densities (HPD) confidence interval. (B) Haplotype network of the *c-mos* nuclear marker. Circle size is proportional to the number of alleles. Sample codes and colours correlate to specimens in Table S1 and in Fig. 1 and Figs. S1–S4. Taxon names correspond to changes proposed in this paper.

The genus is currently comprised of three known species (Uetz & Hošek, 2016): (i) *R. arabicus* Schmidt, 1933 (Holotype FMNH18219; Type locality: Aden, Yemen) is only known from two specimens separated by more than 1,000 km, and both are located more than 2,000 km from the nearest known localities of the other species of the genus. The holotype was collected in Aden, southern Yemen, in 1933. The second specimen was found 80 years later in the Dhofar Governorate, Oman, and released after being sampled for DNA studies (Šmíd et al., 2015). (ii) *R. melanocephalus* (Jan, 1862) (Lectotype MNHG1246.77 designated by Wallach, Williams & Boundy (2014); Type locality: Beirut, Lebanon) was originally described as *Homalosoma melanocephalum* Jan, 1862 and is the most widespread and best-known species of the genus. This Levantine species ranges from the southern Sinai Peninsula through Israel, western Jordan, Lebanon, and Syria to southern Turkey (Franzen & Bischoff, 1995; Disi et al., 2001; Baha El Din, 2006; Avci et al., 2007; Avci et al., 2008; Gasperetti, 1988; Bar & Haimovitch, 2013; Sindaco, Venchi & Grieco, 2013; Werner, 2016). The previously recognized holotype by Günther (1864) from Merom in Israel (specimen BMNH1946.1.3.29) is invalid as Günther’s assignment and name is a synonym of Jan (1862) description. (iii) *R. satunini* (Nikolsky, 1899) (Holotype ZISP9343; Type locality: vicinity of Megri, Armenia) was originally described as *Contia satunini* Nikolsky, 1899, and later assigned as a subspecies of *Oligodon melanocephalus* (Chernov, 1937) or of *R. melanocephalus* (Darevsky, 1970), or as a full species *Rhynchocalamus satunini* (Reed & Marx, 1959). Recently Avci et al. (2015) confirmed its species status. It ranges from south-eastern Turkey eastwards to Iran through the southern Caucasus (Franzen & Bischoff, 1995; Ananjeva et al., 2006; Sindaco, Venchi & Grieco, 2013).

Phylogenetic data on this reclusive genus were, until recently, based on morphology alone—it was classified as an Oriental genus with most of the known species occurring in southern Asia, and at times, it was assigned to the South-east Asian genus *Oligodon* (Boulenger, 1894; Werner, 1905; Chernov, 1937; Bodenheimer, 1944; Haas, 1952; Darevsky, 1970; Amr & Disi, 2011). As no DNA sequences of this genus were available, the squamate phylogeny by Pyron, Burbrink & Wiens (2013) did not include *Rhynchocalamus*, leaving its phylogenetic position unverified. Recently, two studies provided genetic data on the genus (Avci et al., 2015; Šmíd et al., 2015) helping to confirm the phylogenetic affinities. *Rhynchocalamus* was found to be closely related to the genus *Lytorhynchus* within the Western Palearctic colubrid clade (Avci et al., 2015; Šmíd et al., 2015), contradicting the previous hypothesis of an Oriental origin (this relationship, however, was not resolved in the latest squamate phylogeny by Tonini et al., 2016). Although both Avci et al. (2015) and Šmíd et al. (2015) included only four specimens each, the incorporation of molecular data revealed interesting results, such as the first sampling and phylogenetic position of *R. arabicus* (Šmíd et al., 2015) and the elevation of *R. satunini* to species level (Avci et al., 2015). Additional taxonomic changes based on the phylogenetic analyses resulted in the classification of *Rhynchocalamus barani* Olgun et al., 2007, a Turkish endemic, within the new genus *Muhtarophis* Avci et al., 2015, distantly related to *Rhynchocalamus*.

The current status of the recognized species within *Rhynchocalamus*, their relationships and distribution, remain relatively unclear, as no study has sampled all known species from the entire distribution range of the genus. In this work, we explore the phylogenetic relationships within *Rhynchocalamus* by means of a broad sampling coupled with a morphological revision. Using an integrative taxonomic approach (Dayrat, 2005), we seek to produce the most complete phylogeny of *Rhynchocalamus* to date, in order to clarify its systematics, describe a new species from Israel, and elucidate its biogeographical and evolutionary history.

## Materials and Methods

### Taxon sampling, DNA extraction and amplification

In order to resolve the phylogenetic relationships within *Rhynchocalamus*, 29 individuals belonging to all three recognized species were used in the molecular study, including eight sequences from Avci et al. (2015) and Šmíd et al. (2015) that were retrieved from GenBank. In order to evaluate the phylogenetic position of *Rhynchocalamus* within the Western Palearctic colubrid clade, we used 40 sequences of different members retrieved from GenBank. Previous studies have identified *Rhynchocalamus* to be closely related to *Lytorhynchus* (Šmíd et al., 2015) and the newly-described genus *Muhtarophis* (Avci et al., 2015). We therefore additionally sequenced seven *Lytorhynchus* and six *Muhtarophis* individuals. A list of all *Rhynchocalamus* individuals and the members of the Western Palearctic colubrid clade included in the molecular analyses, with their localities and GenBank accession codes, is presented in Table S1. Localities of *Rhynchocalamus* individuals are shown in Fig. 1.

Genomic DNA was extracted from ethanol-preserved tissue samples using the SpeedTools Tissue DNA Extraction kit (Biotools, Madrid, Spain). Individuals were sequenced for the following markers: three mitochondrial gene fragments, ribosomal 12S rRNA and 16S rRNA (*12S* and *16S*, respectively), cytochrome *b* (*cytb*), and the nuclear gene fragment oocyte maturation factor MOS (*c-mos*). Gene fragments were amplified and sequenced for both strands using published primers (as described in detail by Šmíd et al. (2015); when the *cytb* long fragment failed to amplify, we used shorter sequences with the Gludg and Cytb2 primers; Kocher et al., 1989; Palumbi, 1996, respectively).

### Sequence analysis, phylogenetic analyses and hypothesis testing

Chromatographs were checked manually, assembled and edited using Geneious v.7.1.9 (Biomatter Ltd.). For the *c-mos* gene fragment, heterozygous positions were identified and coded according to the IUPAC ambiguity codes in both alleles. Coding gene fragments (*cytb*, *c-mos*) were translated into amino acids and no stop codons were observed, suggesting that the sequences were all functional and were trimmed to start at the first codon position. DNA sequences were aligned for each gene independently using the online version of MAFFT v.7 (Katoh & Standley, 2013) with default parameters (Auto strategy, Gap opening penalty: 1.53, Offset value: 0.0). For the *12S* and *16S* ribosomal fragments we applied the Q-INS-i strategy, in which information on the secondary structure of the RNA is considered. To remove poorly aligned positions of the non-protein-coding *12S* and *16S* we used Gblocks (Castresana, 2000) with low stringency options (Talavera & Castresana, 2007). Inter and intra-specific uncorrected *p*-distances and the number of variable (*V*) and parsimony informative (*Pi*) sites were calculated in MEGA v.7 (Kumar, Stecher & Tamura, 2016) independently for each gene fragment.

We analysed the *Rhynchocalamus* data using four datasets assembled using different species-sets (A, B and C) and different DNA sequences: (i) Species-set A was assembled with the aim of resolving the phylogenetic position of *Rhynchocalamus* within Colubrinae and to obtain dates for some relevant cladogenetic events. Dataset 1 of concatenated mtDNA and nDNA, comprised 82 specimens corresponding to 46 different taxa. Dataset 2, for calibration only, consisted of the concatenated mtDNA and nDNA with a single representative of each mPTP clade of *Rhynchocalamus* (see below for information on the mPTP species delimitation analysis). It comprised 48 specimens corresponding to 46 different taxa; (ii) species-set B was assembled with the aim of resolving the phylogenetic relationships within *Rhynchocalamus*. Dataset 3 of concatenated mtDNA and nDNA included 36 specimens corresponding to six different taxa (*Lytorhynchus* was used to root the tree); (iii) species-set C was assembled with the aim of evaluating the relationships and species boundaries within *Rhynchocalamus*. Dataset 4 of mtDNA haplotypes only included 28 specimens.

Best-fit partitioning schemes and models of molecular evolution were selected with PartitionFinder v.1.1.1 (Lanfear et al., 2012) using the following parameters: branchlengths (linked); models of evolution (beast); model selection (AIC); data blocks (*12S* and *16S* each as a single partition, *cytb* and *c-mos* first and second codon positions of each gene as one partition, and the third codon positions of each gene as another); search scheme (all). A summary of the analyses performed with each dataset, including the different partitions and models is shown in Table S2.

Phylogenetic analyses were performed using maximum likelihood (ML) and Bayesian inference (BI) methods. In both ML and BI analyses, alignment gaps were treated as missing data and the nuclear gene sequences were not phased. Partitions and models for each dataset, with priors, specifications and parameters for each analysis are specified in Table S2. Maximum likelihood trees were estimated in RAxML v.7.4.2 (Stamatakis, 2006) as implemented in raxmlGUI v.1.3 (Silvestro & Michalak, 2012). All ML analyses were performed with a GTR + G model of sequence evolution and 100 replicates. Each inference was initiated with a random starting tree and nodal support was assessed with 1,000 bootstrap pseudoreplicates (Felsenstein, 1985). Bayesian analyses were performed with BEAST v.1.8.2 (Drummond et al., 2012). All BEAST analyses were carried out in CIPRES Science Gateway (Miller, Pfeiffer & Schwartz, 2010), and the .xml file was manually modified to “Ambiguities = true” for the nuclear partition (*c-mos*) to account for variability in the heterozygote positions, rather than treating them as missing data. For all analyses implemented in BEAST, the convergence of runs was assessed by the effective sample size (ESS) values of parameters (>200) using TRACER v.1.6 (Rambaut et al., 2014). LogCombiner and TreeAnnotator (both available in BEAST package) were used to infer the ultrametric tree after discarding 10% as burn-in. Nodes were considered strongly supported if they received ML bootstrap values ≥ 70% and posterior probability (pp) support values ≥ 0.95 (Wilcox et al., 2002; Huelsenbeck & Rannala, 2004).

To identify divergent lineages within *Rhynchocalamus*, putative species boundaries were tested using the multi-rate Poisson Tree Processes (mPTP; Kapli et al., 2016) model, using the webserver (http://mptp.h-its.org/). This is an improved method of the previously published species delimitation method PTP (Zhang et al., 2013). As this analysis relies on single locus data, we reconstructed a ML haplotype concatenated mitochondrial phylogenetic tree as specified above for dataset 4.

A nuclear network was constructed for the nuclear gene *c-mos*. SeqPHASE (Flot, 2010) was used to convert the input files and the software PHASE v.2.1.1 to resolve phased haplotypes (Stephens, Smith & Donnelly, 2001; Stephens & Scheet, 2005). Default settings of PHASE were used, except for phase probabilities, which were set as 0.6. The phased nuclear sequences were used to generate a median-joining network using NETWORKS v.5 (Bandelt, Forster & Röhl, 1999).

In order to test alternative topologies, topological constraints were constructed. We enforced alternative topologies and compared to the unconstrained best ML tree, with the Approximately-Unbiased (AU; Shimodaira, 2002) and Shimodaira-Hasegawa (SH; Shimodaira & Hasegawa, 1999) tests. Per-site log likelihoods were estimated using raxmlGUI v.1.3 (Silvestro & Michalak, 2012) and *p*-values were calculated using CONSEL (Shimodaira & Hasegawa, 2001).

### Divergence time estimates

Unfortunately, no calibration data for *Rhynchocalamus* are currently known, precluding the use of internal calibration points and preventing a direct estimation of the time in our phylogeny. Therefore, for a temporal framework we used calibrations of other members of the Western Palearctic colubrid clade. We used two calibration points previously used by several authors (Nagy et al., 2003; Nagy et al., 2004; Wüster et al., 2008; Pook et al., 2009): (i) *Hemorrhois* divergence between the eastern (*H. ravergieri* and *H. nummifer*) and western (*H. algirus* and *H. hippocrepis*) subgroups occurred after the contact of Africa-Arabia with Eurasia, 16–18 million years ago (Mya; Nagy et al., 2003); we applied a Normal distribution, mean 18, stdev 2 (95% confidence interval of 14.7–21.3 Mya); (ii) *Hierophis* subgroup divergence, including *Eirenis*, 18 Mya according to fossil data (Ivanov, 2002); we applied a Normal distribution, mean 18, stdev 1 (95% confidence interval of 16.4–19.6 Mya). The dataset for the estimation of divergence times in BEAST v.1.8.2 was dataset 2 and comprised the members of the Western Palearctic colubrid clade (species-set A; Table S1) and one representative of each independent mPTP entity of *Rhynchocalamus* (the nuclear genes unphased; Table S1). Partitions, models, priors and parameters are specified in Table S2.

### Morphological material and museum acronyms

The material studied was obtained from the following institutions: [BMNH] Natural History Museum, London, UK; [CAS] California Academy of Sciences, San Francisco, USA; [FMNH] The Field Museum, Chicago, USA; [HUJ] The Hebrew University of Jerusalem, Israel; [MCZ] Museum of Comparative Zoology, Harvard University, USA; [MHNG] Muséum d’histoire naturelle de la Ville de Genève, Switzerland; [MNHN] Muséum national d’Histoire naturelle, Paris, France; [NMW] Naturhistorisches Museum Wien, Austria; [TAU] The Steinhardt Museum of Natural History, Tel Aviv, Israel; [ZDEU] Zoology Department, Ege University, Turkey; [ZFMK] Zoologisches Forschungsmuseum Alexander Koenig, Bonn, Germany; [ZISP] Zoological Institute, St. Petersburg, Russia; [ZMHRU] Zoological Museum of Harran University, Osmanbey, Sanliurfa, Turkey.

Material for the morphological revision of *Rhynchocalamus* comprised 108 alcohol-preserved specimens. In addition, 15 photographed voucher specimens were taxonomically identified and examined (used for meristic, categorical and colouration characters, but not measured), including the lectotype (MHNG1246.77) and previous holotype (BMNH1946.1.3.29) of *R. melanocephalus*; the holotype of *R. satunini* (ZISP9343) and the holotype of *R. arabicus* (FMNH18219, including the pictures published by Šmíd et al., 2015). Localities of the voucher specimens and of the photographic material are presented in Fig. 1. Data from the literature were used to complete missing morphological traits from photographed voucher specimens and to enlarge the sample size for the mensural and meristic comparisons (21 specimens; Nikolsky, 1899; Schmidt, 1933; Reed & Marx, 1959; Darevsky, 1970; Franzen & Bischoff, 1995; Avci et al., 2007; Avci et al., 2008; Šmíd et al., 2015). A list of all examined specimens including their museum accession codes and localities is presented in Table S3.

### Morphological characters

Characters for the morphological analyses were selected based on previous taxonomic studies of the genus *Rhynchocalamus* (Darevsky, 1970; Gasperetti, 1988; Franzen & Bischoff, 1995; Olgun et al., 2007; Avci et al., 2008) and on personal observations. The following mensural characters were taken by the first author (KT; two specimens from the ZMHRU collection were examined by BG) on the right side of each specimen (if bilateral) using Helios callipers with an accuracy to the nearest 0.01 mm and, where necessary, under a stereomicroscope: snout-vent length (SVL), measured from tip of snout to vent; tail length (TL), from vent to tip of tail; pileus length (pilL), measured from tip of snout to posterior margin of parietals; Parietal length (PL); Parietal width (PW); frontal length (FL); frontal width (FW); rostral length (RL); rostral height (RH); rostral width (RW); prefrontal suture length (PFL); internasal suture length (IntNL); eye diameter (EYE); black pattern length (BPL; in *R. melanocephalus* only), from the head to last black dorsal; distance between nostrils (InD); anterior inframaxillars length (AimL); posterior inframaxillars length (PimL).

In addition to the morphometric continuous variables, the following meristic (pholidotic) variables were collected by the same person (KT) using a dissecting microscope: number of preoculars (PreO); number of postoculars (PostO); number of temporal scales (TS); number of post-temporal scales (PTS); number of loreal scales (LS); number of ventrals (VS); number of subcaudal scales (SCS); number of upper labial scales (UL); number of lower labial scales (LL); number of black dorsal scales (BDS; number of black scales at mid-body from between the parietals to the end of colour pattern; in *R. melanocephalus* only); number of lower labials in contact with anterior inframaxillar (InfLC); number of gular scales in a row between posterior inframaxillars (GSI); number of gular scales in a row between posterior inframaxillars and 1st ventral (GS); number of dorsal and temporal scales surrounding the margin of parietals (DST).

Categorical characters describing the degree of size and presence of different scales (not modified in preserved specimens) were: size of the 3rd and 4th upper labial scales (34UL; equal/large) and shape of internasal scales (IntN; triangle/trapezoid).

### Statistical analyses

Statistical analyses were used to investigate if there are differences in the mensural or meristic characters between *R. melanocephalus* and the new species described herein. The 17 mensural and 14 meristic characters were analysed independently, and the two categorical characters were directly used in the description of the new species (see systematic account below).

All the mensural variables were log_10_-transformed before the analyses, and the different datasets were tested for normality using the Shaphiro–Wilk’s test and homogeneity of variances using the Leven’s test; if normality or homogeneity were not present, we used a permutation test. All the statistical analyses were performed in IBM SPSS Statistics 23 (IBM Corp. Armonk, NY, USA). Morphological differences were tested for the meristic variables which presented differences within the assemblage using a one-way ANOVA (for VS, SCS, BDS, DST) and Fisher’s exact probability test (for PostO, LL, InfLC). The mensural traits were tested using one-way ANCOVA (SVL as a covariate for size correction; adult specimens only). As a result of the presence of a single female in the new species, sexual size comparisons could only be tested for *R. melanocephalus* which has an adequate sample size of both males and females, and was tested for each variable as described above.

### Species distribution models

We analysed species distribution models (SDM) using Maxent v.3.3 (Phillips, Anderson & Schapire, 2006) to assess which environmental variables shape *Rhynchocalamus* distribution and whether the species’ ranges could potentially overlap. As a presence-only model, Maxent does not require absence data, which are nearly impossible to obtain for these secretive snakes, and it performs well even with datasets of small sample sizes (Phillips & Dudík, 2008), as is the case with the new species from Israel. Specimen localities were used as the input data. In total, we used 83 records of *R. melanocephalus*, 15 of *R. satunini*, and six of the new species; *R. arabicus* with its two known localities was not included. All three models were developed on the same spatial extent that is shown in Fig. 1. The spatial extent was defined as a 250 km buffer around a polygon encompassing all the localities. The models were based on 19 present-day bioclimatic variables (WorldClim database v.1.4; Hijmans et al., 2005), global land cover data (European Space Agency; http://due.esrin.esa.int/page_globcover.php), and global soil type (FAO; http://www.fao.org/), all at a resolution of 30 arc-seconds. We used ENMTools (Warren, Glor & Turelli, 2010) to calculate Pearson’s correlation coefficient to measure correlation between the climatic variables and retained only variables correlated less than 0.75: altitude, mean diurnal temperature range (BIO2), isothermality (BIO3), temperature seasonality (BIO4), mean temperature of wettest quarter (BIO8), mean temperature of driest quarter (BIO9), mean temperature of coldest quarter (BIO11), precipitation seasonality (BIO15), precipitation of wettest quarter (BIO16), precipitation of driest quarter (BIO17). In Maxent, we used a maximum of 5,000 iterations, 10 replicate runs and 25% of the data was used as training samples. Other settings were left at default. We reclassified the continuous models into binary presence-absence maps using the maximum training sensitivity plus specificity threshold (MTSS). The area under the receiver operating characteristics curve (AUC) was taken as a measure of overall model accuracy.

### Zoobank registration and collection of specimens

The electronic version of this article in Portable Document Format (PDF) will represent a published work according to the International Commission on Zoological Nomenclature (ICZN), and hence the new names contained in the electronic version are effectively published under that Code from the electronic edition alone. This published work and the nomenclatural acts it contains have been registered in ZooBank, the online registration system for the ICZN. The ZooBank LSIDs (Life Science Identifiers) can be resolved and the associated information viewed through any standard web browser by appending the LSID to the prefix http://zoobank.org/. The LSIDs for this publication is: urn:lsid:zoobank.org:pub:1E72A585-C0C3-4835-9466-25E8A7C9FADB. The online version of this work is archived and available from the following digital repositories: PubMed Central and CLOCKSS.

The authors received an ethical permission (Ege University Animal Experiments Ethics Committee, 2010#43) and special permission (2014#51946) for field studies from the Republic of Turkey, Ministry of Forestry and Water Affairs. Genetic and morphological analyses of specimens from other localities were based on museum collections and no specimens were collected for this work. All efforts were made to minimize animal suffering.

## Results

### Molecular analyses

The phylogenetic results within the Western Palearctic colubrid clade (dataset 1; Fig. S1), for 82 sequences of 46 taxa, agree with the phylogenetic placement of the new genus *Muhtarophis* as distinct from *Rhynchocalamus*, although its phylogenetic position within the clade remains unresolved (no support in the ML analysis, but strong support in the BI analysis). *Wallaceophis* is sister to a clade comprising *Rhynchocalamus* and *Lytorhynchus*, though with no support in either ML or BI analyses. Both *Rhynchocalamus* and *Lytorhynchus* are recovered as monophyletic sister genera with strong support.

The phylogenetic relationships within *Rhynchocalamus* (dataset 3; Fig. 2A) are based on 29 specimens of the three known species within the genus, comprising one sample of *R. arabicus*, four of *R. satunini* and 24 samples of *R. melanocephalus* (including four samples of the new species described herein; Table S1). This dataset included mitochondrial gene fragments of *12S* (618 bp; *V* = 94; *Pi* = 73), *16S* (510 bp; *V* = 42; *Pi* = 36), *cytb* (1,092 bp; *V* = 257; *Pi* = 210), and nuclear gene fragment of *c-mos* (408 bp; *V* = 4; *Pi* = 3), totalling 2,628 bp. Genetic distances for the mitochondrial markers are presented in Table S1.

*Rhynchocalamus* (Fig. 2A) is divided into four separate lineages. The monophyly of the distinct lineage of *R. satunini* from Turkey and Iran is strongly supported. Within this species, the three specimens from Turkey form an inner group, while the specimen from Iran is relatively distant. This species is more genetically diverse than the other species (*12S*: 1%; *16S*: 0.4%; *cytb*: 1.8%; Table S1). The sole specimen of *R. arabicus* from Oman is genetically distinct. The results reveal that *R. melanocephalus* is paraphyletic, as the southern Israeli lineage from the Negev Mountain in Israel (the new species described herein, see Systematic account section below) is phylogenetically more closely related to *R. arabicus* from Oman than to the geographically adjacent populations of *R. melanocephalus* from the Negev region in Israel and northwards to Turkey (Fig. 1). In addition, within the *R. melanocephalus* lineage, one sample from Mt. Hermon in Israel (HUJ.R20967) is phylogenetically distinct from the others.

The *c-mos* nuclear network (Fig. 2B) reveals a pattern of incomplete lineage sorting. Allele sharing is present between the newly-described species from the Negev Mountain in Israel and the Negev population of *R. melanocephalus*. This is based, however, on only two samples from the Negev Mountain population (the other two samples from this population failed to amplify). Allele sharing also occurs between *R. arabicus* from Oman and *R. satunini* from Iran and Turkey.

The mPTP species delimitation approach recognized six distinct entities within *Rhynchocalamus* (Fig. S2): two discrete entities within *R. satunini* (the specimen from Iran as distinct from the three specimens from Turkey); a distinct *R. arabicus*; and three entities within *R. melanocephalus*–the southern lineage from the Negev Mountain in Israel is distinct (the new species described below), recovered as sister species to *R. arabicus*, separated from the rest of the *R. melanocephalus* specimens, and a sister relationship between the sole sample from Mt. Hermon and the rest of the samples from the Negev area in Israel northwards to Turkey.

We performed a topology test in order to better understand the relationships recovered in our analyses, by forcing the monophyly of *R. melanocephalus* and the new species described herein. The results of this test (AU: *p* < 0.0001; SH: *p* = 0) reject the hypothesis that *R. melanocephalus* and the new species described herein form a clade.

Divergence time estimates for the Western Palearctic colubrid clade are presented in Fig. S3, and near the relevant nodes for *Rhynchocalamus* in Fig. 2A. We selected six representatives of *Rhynchocalamus* to be used in the dating analysis according to the mPTP species delimitation method (Figs. S2–S3; Table S1). Our results, based on dataset 2, indicate that *Rhynchocalamus* split from *Lytorhynchus* around 26.4 Mya (95% HPD: 21.6–31.9 Mya). *Rhynchocalamus* started diverging with the split of *R. satunini* during the Middle Miocene ca. 15.5 Mya (95% HPD: 12–19.4 Mya). The divergence of *R. melanocephalus* is estimated to have occurred around 11.3 Mya (95% HPD: 8.7–14.4 Mya), with further radiation during the Pliocene, ca. 3.5 Mya (95% HPD: 1.7–5.8 Mya). The separation between *R. arabicus* and the new species described herein from the Negev Mountain in Israel appears to have occurred approximately 9.5 Mya (95% HPD: 7–12.5 Mya).

### Morphological analyses

The morphological database comprised 118 specimens of *R. melanocephalus* (six of which belong to the new species described herein, see below), 15 specimens of *R. satunini* and two specimens of *R. arabicus*. Descriptive statistics for all 33 variables included in the analysis are presented in Table 1.

**Table 1:** Descriptive statistics for all variables examined for males and females of *Rhynchocalamus*. Two populations of *R. melanocephalus* are presented (Southern population from the Negev region in Israel; Northern population from the Mediterranean ecoregion). Mean ± standard deviation and the range (min–max) are given. Mensural variables were taken from adult specimens only and are presented in millimetres. Bilateral meristic characters are presentedt with the left/right sides. Abbreviations of variables as explained in ‘Materials and Methods’. Taxon names correspond to changes proposed in this paper.

Variable	*R. arabicus* (*n* = 2)			*R. melanocephalus*					
		*R. dayanae* sp. nov.		Southern population (*n* = 8)		Northern population (*n* = 94)		*R. satunini*	
		Males (*n* = 5)	Females (*n* = 1)	Males (*n* = 7)	Females (*n* = 1)	Males (*n* = 64)	Females (*n* = 30)	Males (*n* = 5)	Females (*n* = 6)
SVL	278–289	368.9 ± 75.4 (259.8–432.1)	278.8	335.1 ± 45.8 (266.8–391.2)	344.4	351.7 ± 62.2 (189.48–464.7)	376.9 ± 70.2 (257.7–499.2)	272.9 ± 72 (198–341.5)	267.5 ± 56.7 (185–310)
TL	49	78.1 ± 16.5 (59.2–94.1)	59.2	80.1 ± 11.3 (64.3–94.97)	86.3	82.6 ± 17.3 (11.05–109.1)	78.4 ± 18.8 (26–105.3)	54.3 ± 16.6 (36–68.4)	52.7 ± 15.6 (31–68)
pilL	–	8.1 ± 0.8 (7.05–8.82)	7.61	8 ± 0.7 (7.06–9.32)	7.46	8.2 ± 0.8 (6.42–10.03)	8 ± 0.6 (6.78–9.52)	7.4 ± 0.5 (6.84–7.9)	7.7 ± 1.3 (6.78–8.55)
RL	–	1.6 ± 0.3 (1.28–1.92)	1.41	1.4 ± 0.1 (1.23–1.62)	1.51	1.5 ± 0.2 (1.1–2.2)	1.4 ± 0.2 (0.99–1.8)	2.21	1.75
RH	–	1.4 ± 0.3 (1.15–1.66)	1.11	1.7 ± 0.2 (1.39–1.85)	–	1.84 ± 0.3 (1.22–2.53)	1.76 ± 0.3 (1.38–2.34)	1.8 ± 0 (1.78–1.87)	1.9 ± 0.3 (1.66–2.11)
RW	–	2.3 ± 0.6 (1.79–2.88)	1.94	2.3 ± 0.2 (1.95–2.48)	–	2.5 ± 0.3 (1.75–3.39)	2.3 ± 0.3 (1.74–3.08)	2.1 ± 0.1 (2.05–2.18)	2.5 ± 0.1 (2.46–2.61)
FL	–	2.9 ± 0.2 (2.66–3.18)	2.46	3 ± 0.4 (2.55–3.58)	3.06	3.1 ± 0.3 (2.4–3.98)	2.9 ± 0.3 (2.17–3.36)	2.6 ± 0.1 (2.48–2.75)	2.4 ± 0.6 (2.02–2.8)
FW	–	2.6 ± 0.4 (2.02–2.84)	2.39	2.5 ± 0.1 (2.32–2.65)	2.18	2.5 ± 0.3 (1.99–3.11)	2.4 ± 0.3 (1.95–3.06)	2.1 ± 0.1 (2.03–2.2)	2.2 ± 0 (2.15–2.18)
PL	–	3.6 ± 0.4 (3.11–3.86)	3.41	3.7 ± 0.4 (3.19–4.25)	3.22	3.7 ± 0.4 (2.88–4.77)	3.6 ± 0.3 (3.02–4.11)	3.75	3.9
PW	–	4.2 ± 0.5 (3.73–4.71)	4.11	4.8 ± 0.6 (4.03–5.49)	5.93	4.8 ± 0.6 (3.26–6.35)	4.6 ± 0.5 (3.68–5.47)	3.7 ± 0.7 (3.28–4.47)	3.9 ± 0.8 (3.3–4.45)
IntNL	–	0.5 ± 0.1 (0.41–0.68)	0.66	0.6 ± 0.1 (0.39–0.69)	0.52	0.5 ± 0.1 (0.21–0.77)	0.4 ± 0.2 (0.11–0.85)	0.32	0.35
PFL	–	0.8 ± 0.1 (0.71–0.92)	0.62	0.8 ± 0.2 (0.64–1.27)	0.54	0.6 ± 0.2 (0.18–1.08)	0.6 ± 0.2 (0.21–1.02)	0.68	1.05
EYE	–	1.5 ± 0.2 (1.31–1.61)	1.36	1.7 ± 0.3 (1.31–2.11)	1.14	1.5 ± 0.2 (1.19–1.91)	1.4 ± 0.2 (1.02–1.7)	1.3 ± 0.3 (1.05–1.66)	1.29 ± 0 (1.27–1.3)
BPL	–	19.8 ± 2.7 (16.32–22.95)	17.91	20 ± 3.1 (14.78–23.24)	17.32	15.4 ± 2.5 (10.77–23.25)	14.2 ± 1.4 (11.05–17.05)	–	–
InD	–	1.7 ± 0.4 (1.32–2.11)	1.52	2.1 ± 0.2 (1.92–2.46)	1.64	2.3 ± 0.3 (1.59–3.24)	2.2 ± 0.3 (1.56–3.04)	2.1 ± 0.2 (2–2.28)	2.2
AimL	–	2.4 ± 0.3 (2.04–2.64)	2.35	2.2 ± 0.3 (1.87–2.64)	–	2.2 ± 0.4 (1.4–3.06)	2.1 ± 0.3 (1.55–2.44)	2 ± 0.03 (2–2.08)	1.6 ± 0.5 (1.24–1.98)
PimL	–	1.9 ± 0.1 (1.79–1.92)	1.57	1.6 ± 0.2 (1.34–1.92)	–	1.8 ± 0.3 (1.13–2.37)	1.6 ± 0.3 (1.18–2.44)	1.4 ± 0.3 (1.16–1.78)	1.3 ± 0.6 (0.88–1.75)
PreO	1/1	1/1	1/1	1/1	1/1	1/1	1/1	1/1	1/1
PostO	1/1	2/2 (80%)	2/2	1/1 (71%)	1/1	1/1 (98%)	1/1	1/1	1/1
TS	1/1	1/1	1/1	1/1	1/1	1/1	1/1	1/1	1/1
PTS	1/1	1/1 (88%)	1/1	2/2 (57%)	1/1	2/2 (84%)	2/2 (83%)	1/1 (90%)	1/1 (63%)
LS	1/1	1/1	1/1	1/1	1/1	1/1 (89%)	1/1 (97%)	1/1	1/1 (90%)
VS	240	210 ± 13.1 (198–229)	188	188.4 ± 14.9 (168–209)	–	195.5 ± 12.3 (178–234)	198.8 ± 15.4 (164–235)	203.5 ± 2.4 (201–206)	221.5 ± 4 (215–226)
SCS	71-81	59.2 ± 3.3 (54–62)	58	60 ± 4 (52–65)	53	58.8 ± 6.6 (29–69)	56.7 ± 8.4 (29–68)	60.3 ± 2.9 (58–64)	59.2 ± 3.6 (53–64)
UL	6/6	6/6	6/6	6/6	6/6	6/6	6/6	7/7 (80%)	7/7
LL	8/8	8/8	8/8	7/7	7/7	7/7 (89%)	7/7 (85%)	8/8 (60%)	8/8
BDS	–	10–16	9	11–16	13	5–14 (5–9, 82%)	5–12 (5–9, 89%)	–	–
InfLC	–	4	4	3 (79%); 4 (21%)	3	3 (73%); 4 (27%)	3 (74%); 4 (26%)	4 (88%)	3 (50%); 4 (50%)
GSI	–	2	2	2	2	1 (21%); 2 (79%)	1 (27%); 2 (73%)	1 (75%)	2 (60%)
GS	–	3	3	4-5	4	2–6 (3–5, 97%)	3–5 (4–5, 86%)	3–4	4–5
DST	12–13	10–12	12	9.9 ± 0.9 (9–11)	11	10.8 ± 1 (9–13)	10.4 ± 1.2 (8–13)	10–12	12–13
34UL	Large	Large	Large	Large	Large	Large (77%)	Large (79%)	Large	Large
IntN	Triangle (50%)	Trapezoid (60%)	Trapezoid	Trapezoid (83%)	Trapezoid	Trapezoid (64%)	Trapezoid (75%)	Trapezoid (75%)	Triangle (60%)

The morphological characteristics of the four lineages of *Rhynchocalamus*, comprising 17 mensural, 14 meristic and two categorical traits (Table 1) are as follows: cylindrical body with 15 smooth dorsal scales rows; a small head indistinct from the neck; reduced maxillary dentition with 6–8 maxillary teeth, the posterior ones being long; a rostral shield enlarged, pointed backwards and wedged between the internasals; nostril situated in an undivided elongated nasal scale; small eyes with round pupils; loreal scale usually present in all species (absent in 8.5% of *R. melanocephalus*); 3rd and 4th upper labial scales usually larger than the 1st and 2nd (equal in size in 19% of *R. melanocephalus*) and are in contact with the eye; one preocular scale; one postocular scale in *R. satunini*, *R. arabicus* and *R. melanocephalus* (the latter comprises three specimens with two postocular scales on either side), two postocular scales in the new species described herein; one temporal scale, and between one or two post-temporals (mainly two in *R. melanocephalus* and one in *R. satunini*, *R. arabicus* and the new species); seven upper labial scales in *R. satunini*, six in the others; the shape of the 1st upper labial scale varies between square and trapezoid (34% and 66%, respectively), and that of the 2nd scale is mainly square; mostly seven lower labial scales in *R. melanocephalus* (11% with eight scales), eight in the others, including in the new species described below; mostly four lower labial scales in contact with the anterior inframaxillars in *R. satunini* and the new species described below, mostly three in *R. melanocephalus* (74%); single gular scale in contact with anterior inframaxillars, situated between the posterior inframaxillars; posterior inframaxillars are separated by 1–2 gular scales (22% and 78%, respectively); 2–8 gular rows separate the posterior inframaxillars from the 1st ventral (3–5, 97%); 5–16 black dorsal scales at mid-body in *R. melanocephalus*, 10–16 in the new species described below; number of dorsal and temporal scales surrounding the margin of parietals is 8–15; 164–240 ventrals; anal and subcaudal scales divided; 29–81 subcaudal scales.

### Statistical analyses of morphological data

The results of the sexual dimorphism analysis of *R. melanocephalus* using one-way ANOVA showed that there are no significant differences in body-size between males and females (SVL; *P* = 0.06). The one-way ANCOVA results (with SVL as a covariate for size correction) for the remaining mensural characters showed that significant sexual size dimorphism is present for the following 13 characters, for most the males have larger sizes (*P* < 0.047 for all variables; Table S4): pileus length (pilL), rostral length (RL), rostral height (RH), rostral width (RW), frontal length (FL), frontal width (FW), parietal length (PL), frontal width (PW), eye diameter (EYE), black pattern length (BPL), distance between nostrils (InD), anterior inframaxillars length (AimL), and posterior inframaxillars length (PimL). The remaining three mensural characters did not show significant differences between the sexes (*P* > 0.17 for all three variables). The results of the one-way ANOVA and the Fisher’s exact probability test of seven meristic characters (VS, SCS, BDS, DST and PostO, LL, InfLC, respectively) showed no sexual dimorphism (*P* > 0.07 and 0.17 for all variables, respectively; Table S4).

As a result of the presence of sexual size dimorphism within *R. melanocephalus*, and due to the presence of only one female of the new species described herein, differences between the two species were only tested among male specimens for each variable as described above. Statistical tests did not detect significant differences in most of the mensural and meristic traits between the new species and *R. melanocephalus* (*P* > 0.068 for all characters; Table S4). For the mensural traits significant differences were found for the following four characters (Table S4): (1) Rostral height (RH; *P* = 0.003), indicating that the new species has a lower rostral shield than *R. melanocephalus* (1.15–1.66 mm *vs.* 1.22–2.53 mm, respectively); (2) Parietal width (PW; *P* = 0.035), indicating that the new species has a narrower head width than *R. melanocephalus* (3.73–4.71 mm *vs.* 3.26–6.35 mm, respectively); (3) Distance between nostrils (InD; *P* < 0.0001), indicating that the new species has a shorter distance than present in *R. melanocephalus* (1.32–2.11 mm *vs.* 1.59–3.24 mm, respectively); (4) Black pattern length (BPL; *P* = 0.001), indicating that the new species has relatively longer dorsal black colour pattern than *R. melanocephalus* (16.32–22.95 mm *vs.* 10.77–23.25 mm, respectively). The latter finding, in contrast to the other results, is not significant when the new species is compared to the Negev population of *R. melanocephalus* (Localities 6–12 in Fig. 1; *P* = 0.307; 16.32–22.95 mm *vs.* 14.78–23.24 mm, respectively). For the meristic variables, two characters showed significant differences between the two assemblages (Table S4): number of ventrals (VS; *P* = 0.015), indicating that the new species has a higher number of ventrals than *R. melanocephalus* (198–229 *vs.* 178–234, respectively); and the number of black dorsal scales (BDS; *P* < 0.0001), corresponding to the mensural trait of black pattern length (BPL), which was also significant, indicating that the new species has more back dorsal scales than *R. melanocephalus* (10–16 *vs.* 5–14, respectively). As with the BPL character, this finding is not significant when the new species is compared to the Negev population of *R. melanocephalus* (Localities 6–12 in Fig. 1; *P* = 0.872; 10–16 *vs.* 11–16, respectively). Three meristic characters (PostO, LL, InfLC) showed significant results between the two species for each variable (*P* < 0.006; Table S4), indicating that the new species has a significantly higher number of postocular scales (two *vs.* one in *R. melanocephalus*), higher number of lower labials (eight *vs.* mostly seven in *R. melanocephalus*), and a higher number of lower labials in contact with anterior inframaxillar (four *vs.* mostly three in *R. melanocephalus*).

### Potential distribution of *Rhynchocalamus*

The distribution models of *R. melanocephalus*, *R. satunini* and the new species from southern Israel had either excellent or good predictive accuracy (following Araújo et al., 2005) with AUC averaged over ten replicate runs being 0.977 ± 0.015, 0.835 ± 0.071 and 0.999 ± 0.001, respectively. The potential distributions predicted for the presence of the three species show overlapping distributions (Fig. S4). The range of *R. satunini* stretches from Iran westwards across the southern Caucasus to southern Turkey where it overlaps with *R. melanocephalus* which spans from southern Turkey along the eastern Mediterranean coast to the Sinai Peninsula. In its southernmost range *R. melanocephalus* overlaps with the new species which is restricted mostly to southern Israel. The two latter species have a potential presence in the northern Sinai Peninsula. The main environmental variables that contributed most to the predicted distributions were BIO4 (59.2%), soil type (16.5%), BIO16 (15.2%) for *R. melanocephalus*; soil type (35.9%), BIO3 (29%), BIO4 (16.3%) for *R. satunini*; and soil type (51.8%), BIO4 (24.6%), BIO15 (20.4%) for the new species.

### Systematic account

The findings from this study identify a discrepancy in the known systematics of the genus *Rhynchocalamus* and, within it, in the taxonomy of *R. melanocephalus*—in suggesting a separation between the populations of *R. melanocephalus* in Israel. This species is separated into two lineages: one from the Negev region in Israel northwards to Turkey (including the lectotype of *R. melanocephalus*), and the other, southern, limited to the Negev Mountain region. These results are evident in both the genetic analyses (using three mitochondrial and one nuclear gene fragments; Fig. 2), and the morphological comparisons (see results above and comparison below; Table 1). We therefore describe the southern population of *R. melanocephalus* from the Negev Mountain region in Israel as a new species:

**Table:** 

Family Colubridae
Genus *Rhynchocalamus* Günther, 1864
*Rhynchocalamus dayanae* **sp. nov.**
urn:lsid:zoobank.org:act:8FC77FE5-1262-4631-AC2D-F202B5A9003C
(Tables 1–2, Tables S1–S4; Figs. 1–6, Figs. S1–S4).

*Rhynchocalamus melanocephalus* Barash & Hoofien, 1956: 144 (part.); Arbel, 1984: 132 (part.); Bouskila & Amitai, 2001: 258 (part.); Bar & Haimovitch, 2011: 166; 2013:170 (part.); Werner, 2016: 271 (part.).

**Figure 3:**
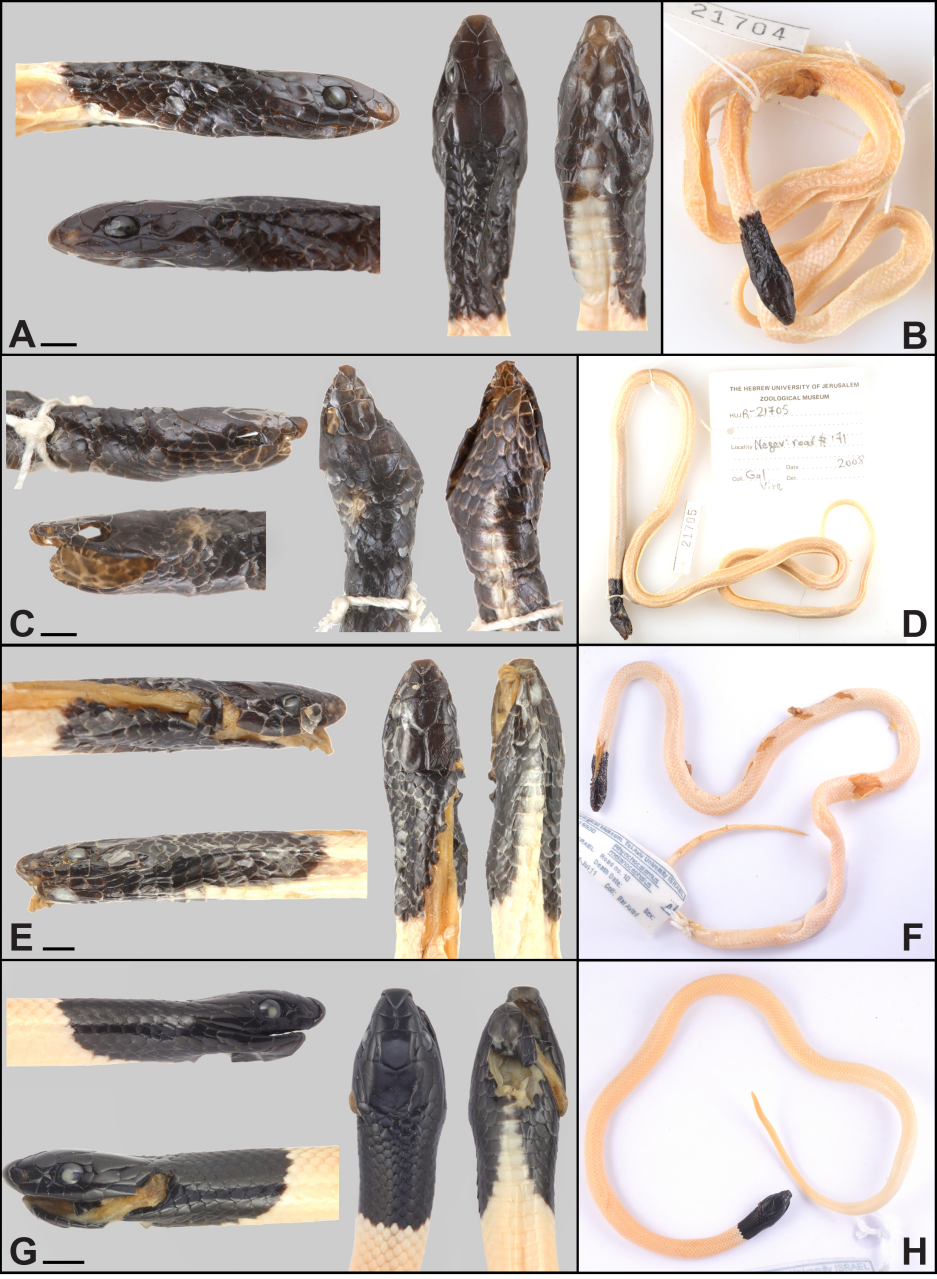
Type series of *Rhynchocalamus dayanae* sp. nov. Head and habitus (photos by Oz Rittner). (A, B) Holotype HUJ.R21704; (C, D) Paratype HUJ.R21705 (no skull); (E, F) Paratype TAU.R15930; (G, H) Paratype TAU.R17093. Scale bars = 2.5 mm.

*Holotype.* HUJ.R21704, Adult male, collected from road no. 40 near Nafha Prison, Negev Mountain, Israel, 30.7317N 34.7709E WGS84, 700 m above sea level (a.s.l.) on the 21st of June 2008 by Gal Vine (Fig. 3).

*Paratypes.* TAU.R15930 (Adult male, collected from road no. 10, Nahal Batur, 30.3922N 34.5918E, 700 m a.s.l., by Aviad Bar on the 11th of July 2011); TAU.R17093 (Sub-adult male, collected from Mitzpe Ramon, 30.6108N 34.8029E, 850 m a.s.l., by Maya Spector on the 26th of October 2014); HUJ.R21705 (Adult male, collected from road no. 171, 30.5011N 34.5884E, by Gal Vine on the 3rd of June 2008). All paratypes (Fig. 3) were collected in the Negev Mountain area, Israel, and were included in the molecular analysis (Table S1).

*Other material.* HUJ.R22021 (Adult female, collected from road no. 40, Mizpe Ramon quarries, 30.6306N 34.8051E, 810 m a.s.l., by Gal Vine on the 23rd of June 2010); HUJ.R22023 (Adult male, collected from road no. 171, 30.501N 34.577E, 900 m a.s.l., by Gal Vine on the 26th of May 2010).

*Etymology.* The specific epithet, “*dayanae*,” is named in honour of Professor Tamar Dayan, director of the Steinhardt Museum of Natural History at Tel Aviv University and curator of the Terrestrial Vertebrate Collection. This naming of the new species constitutes a special recognition of Professor Dayan by two of her former students (KT and SM) to acknowledge her immense contribution to the conservation of Israeli fauna, and her efforts in establishing the National Natural History Museum at Tel Aviv University, and in promoting taxonomy, conservation and ecology studies in Israel.

**Table 2:** Morphological variables for the type series of *R. dayanae* sp. nov. Mensural variables are presented in millimetres. Bilateral meristic characters are presented with the left/right sides. Abbreviations of variables as explained in ‘Materials and Methods.’

Variable	Holotype HUJ.R21704	Paratype HUJ.R21705	Paratype TAU.R15930	Paratype TAU.R17093
	Adult, ♂	Adult, ♂	Adult, ♂	Sub-adult, ♂
SVL	259.8	432.1	384.7	213.1
TL	59.2	89.5	94.1	47.1
pilL	7.05	8.18	8.82	6.92
RL	1.42	1.28	1.59	1.39
RH	1.15	–	1.66	1.58
RW	1.79	–	2.08	1.81
FL	2.66	3.18	2.84	2.46
FW	2.02	2.84	2.73	2.32
PL	3.11	3.85	3.77	3.21
PW	3.73	–	4.17	4.09
IntNL	0.41	0.61	0.68	0.33
PFL	0.78	0.71	0.92	0.59
EYE	1.31	–	1.61	1.21
BPL	16.32	22.95	19.81	13.62
InD	1.32	–	1.75	1.71
AimL	2.04	–	2.59	2.27
PimL	1.84	–	1.79	–
PreO	1/1	1/1	1/1	1/1
PostO	2/1	2/2	2/2	2/1
TS	1/1	1/1	1/1	1/1
PTS	1/1	2/1	1/1	1/1
LS	1/1	1/1	1/1	1/1
VS	201	229	218	198
SCS	62	60	62	54
UL	6/6	6/6	6/6	6/6
LL	8/8	–/8	8/–	–/8
BDS	12	16	12	10
InfLC	4/4	4/4	4/4	4/4
GSI	2	2	2	–
GS	3	3	3	–
DST	11	12	11	10
34UL	Large	Large	Large	Large
IntN	Triangle	Trapezoid	Trapezoid	Trapezoid

**Figure 4:**
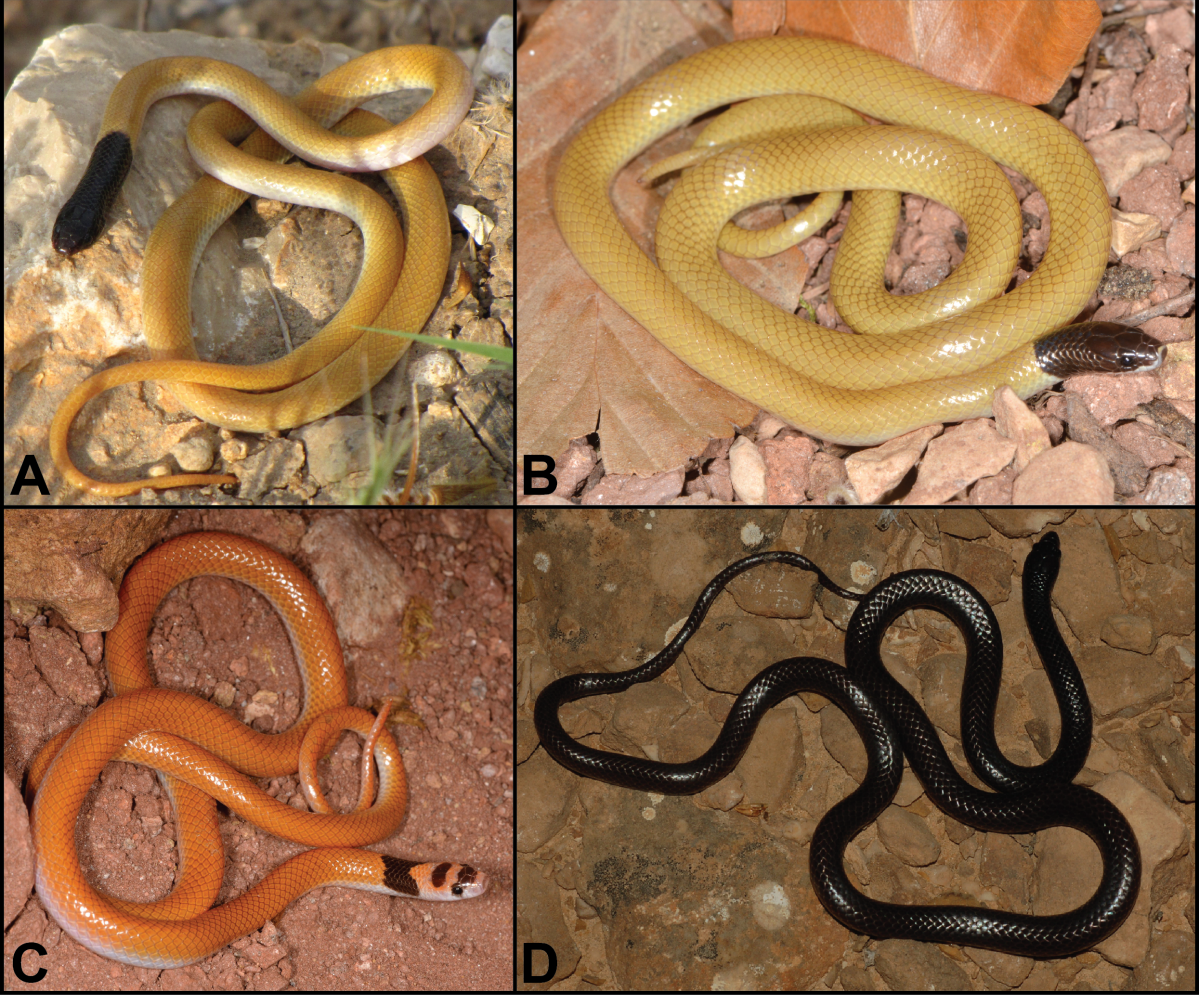
Habitus comparisons of *Rhynchocalamus* taxa. Dorsal view. (A) *R. dayanae* **sp. nov.** (unvouchered specimen; Road no. 40, near Mitzpe Ramon, Negev Mountain, Israel; photo by Simon Jamison); (B) *R. melanocephalus* (ZMHRU2007-69; Tartus, Syria; photo by Bayram Göçmen); (C) *R. satunini* (ZMHRU2015/0; Artuklu, Mardin province, Turkey; photo by Bayram Göçmen); (D) *R. arabicus* (CN4780; Wadi Ayoun, Dhofar Governorate, Oman; photo by Gabriel Martínez).

**Figure 5:**
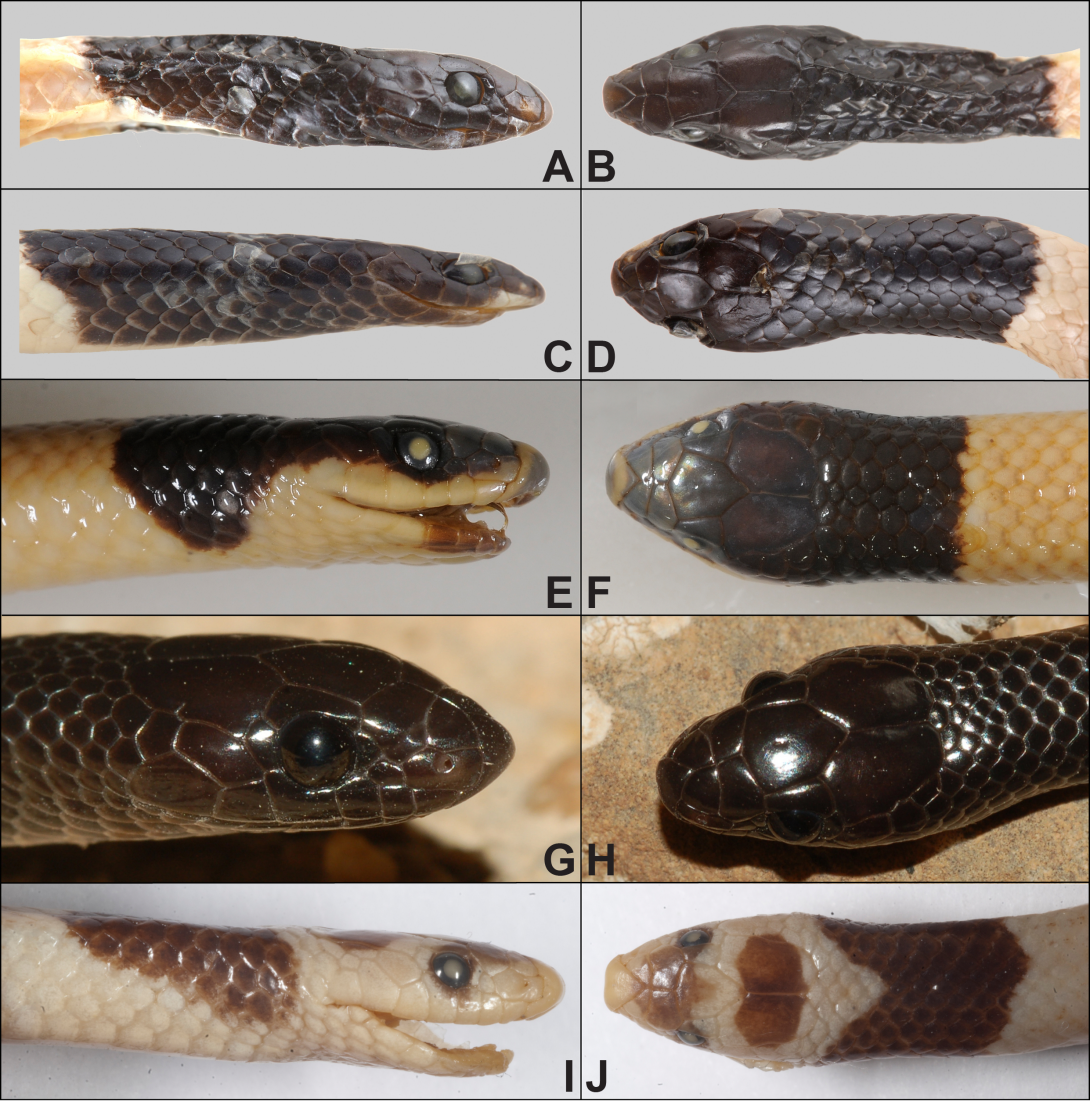
Head comparisons of *Rhynchocalamus* taxa. Lateral and dorsal views. (A, B) *R. dayanae* **sp. nov.** (Holotype, HUJ.R21704; photos by Oz Rittner); (C, D) *R. melanocephalus* (Negev population; HUJ.R22058; photos by Oz Rittner); (E, F) *R. melanocephalus* (Mediterranean ecoregion population; BMNH1946.1.3.29; photos by Salvador Carranza); (G, H) *R. arabicus* (CN4780; photos by Gabriel Martínez); (I, J) *R. satunini* (ZISP17098; photos by Daniel Melnikov).

**Figure 6:**
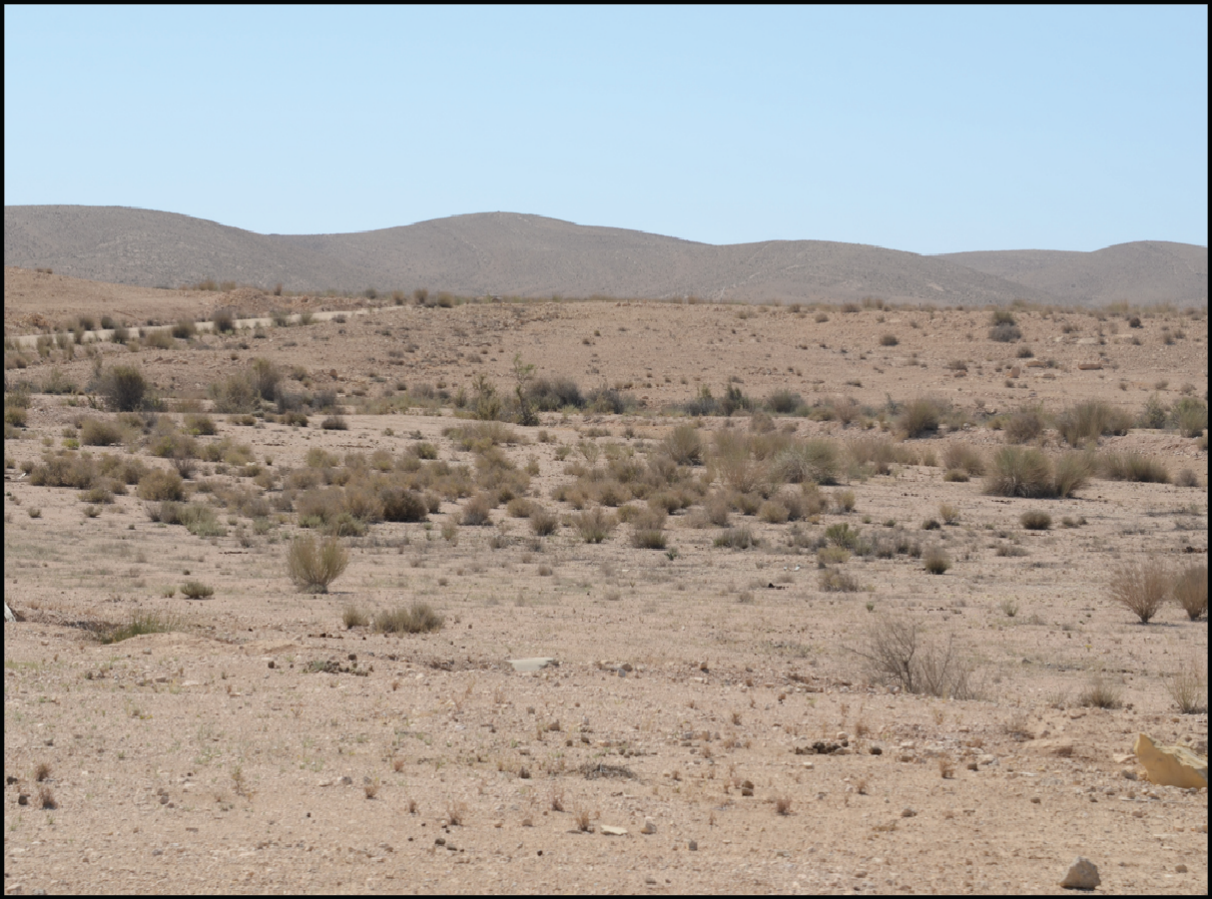
Habitat of *R. dayanae* sp. nov The Negev Mountain region, road no. 171, Israel (the general area where the type material was collected).

*Diagnosis.* A new species of *Rhynchocalamus* from the Negev Mountain in southern Israel characterized by the combination of the following characters: (1) SVL 259.8–432.1 mm in adults; (2) tail length 59.2–94.1 mm in adults; (3) loreal scale present; (4) 3rd and 4th upper labial scales large and in contact with the eye; (5) one preocular scale; (6) usually two postocular scales on either side; (7) one temporal scale; (8) one post-temporal; (9) six upper labial scales; (10) eight lower labial scales; (11) four lower labial scales in contact with the anterior inframaxillars; (12) single gular scale in contact with anterior inframaxillars, situated between the posterior inframaxillars; (13) 2–8 gular rows separate the posterior inframaxillars from the 1st ventral; (14) 10–16 black dorsal scales in mid-body (Figs. 3–5); (15) 10–12 dorsal and temporal scales surrounding the margin of parietals; (16) 188–229 ventrals; (17) anal and subcaudal scales divided; (18) 54–62 subcaudal scales.

*Differential diagnosis. Rhynchocalamus dayanae* **sp. nov.** differs from the other species of the genus in its head scalation and its head and dorsal colour pattern (Table 1; Figs. 4–5). It differs from its phylogenetically closely-related taxon, *R. arabicus* from Yemen and Oman, in its lower number of ventrals (188–229 *vs.* 240 in *R. arabicus*) and subcaudals (54–62 *vs.* 71–81 in *R. arabicus*), and the presence of two postocular scales (one in *R. arabicus*). The overall black colouration of *R. arabicus* is an additional differentiation from the solely black head and first dorsal scales of *R. dayanae* **sp. nov.** It further differs by a genetic distance of 5.9%, 4.2%, and 10% in the mitochondrial *12S*, *16S*, and *cytb* genes, respectively (Table S1).

*Rhynchocalamus dayanae* **sp. nov.** differs from the geographically distant species, *R. satunini* from Turkey eastwards to Iran, in the lower number of upper labials (6 *vs*. 7 in *R. satunini*), and the presence of two postocular scales (one in *R. satunini*). The head colouration of *R. satunini* is not uniform black, but features two black patches on a whitish background around the prefrontal scales and the parietals, and a black band around the neck that does not reach the ventrals (the rostral shield, nasals, loreals, upper and lower labials, temporal, and ventrals are whitish). It further differs by a genetic distance of 7.3%, 5.4%, and 11.9% in the mitochondrial *12S*, *16S*, and *cytb* genes, respectively (Table S1).

*Rhynchocalamus dayanae* **sp. nov.** differs from *R. melanocephalus* (including its Negev population) by the combination of a higher number of lower labials, eight (100%) *vs.* seven (90%); higher number of lower labials in contact with the anterior inframaxillars, four (100%) *vs.* three (74%); the presence of two postocular scales (84% *vs.* 3%); a higher number of ventrals (198–229 *vs.* 178–234); lower rostral shield (1.15–1.66 mm *vs.* 1.22–2.53 mm); a narrower head width (3.73–4.71 mm *vs.* 3.26–6.35 mm); a narrower distance between nostrils (1.32–2.11 mm *vs.* 1.59–3.24 mm). *Rhynchocalamus dayanae* **sp. nov.** differs from the northern, Mediterranean populations of *R. melanocephalus* (from central Israel northwards) by the larger extent of the black dorsal pattern (BDS, 13 ± 2.1 (10–16) *vs.* 7.8 ± 1.8 (5–14); BPL, 19.8 ± 2.7 (16.32–22.95) mm *vs.* 15.4 ± 2.5 (10.77–23.25) mm, respectively) and black gulars (absent in the northern *R. melanocephalus*). *Rhynchocalamus dayanae* **sp. nov.** further differs from all populations of *R. melanocephalus* by a genetic distance of 6.7%, 4%, and 10.2% in the mitochondrial *12S*, *16S*, and *cytb* genes, respectively (Table S1).

*Description of the holotype.* Adult male (HUJ.R21704) (Fig. 3). Snout-vent length 259.8 mm, tail length 59.2 mm. Six to eight maxillary teeth, the posterior teeth are long and strong, broad at the base with an impression but without a longitudinal groove, palatine teeth absent, mandibular teeth slightly longer anteriorly than posteriorly. Body cylindrical and slender, almost consistently thick from the neck to the tail base. Head small and relatively narrow (Pileus length 7.05 mm; Pileus length/parietal width = 1.9), indistinct from the neck, with pointed oblique shape at the anterior side. Head scales, from the rostral to the posterior margin of parietals, including the temporals and post-temporals, not keeled. Rostral scale enlarged, extending backwards, intruding between the internasals, with a length of 1.42 mm, height of 1.15 mm and width of 1.79 mm. Rostral bordered by two upper labials, two nasals and two internasals. Nostrils situated on undivided nasal scales with a distance of 1.32 mm between them. Internasals of triangular shape. The internasal suture (length 0.41 mm) is almost half the length of the prefrontal suture (length 0.78 mm). A square-shaped loreal scale at either side in contact with 2nd and 3rd upper labials. The eyes are small with circular pupil of 1.31 mm in diameter. One preocular scale on both sides. Two postocular scales on the left side, and one on the right side. Frontal scale situated between the supraoculars with a length of 2.66 mm and width of 2.02 mm. Six squarish upper labials, 3rd and 4th in contact with eye. Two large parietal scales with a length of 3.11 mm and width of 3.73 mm. One temporal and one post-temporal scale on each side. Eight lower labials. Four pairs of lower labials in contact with anterior inframaxillars on each side. One gular scale positioned between posterior inframaxillars and in contact with anterior inframaxillars. Anterior and posterior inframaxillars lengths are 2.04 mm and 1.84 mm, respectively. Eleven dorsal and temporal scales surrounding the posterior margin of the parietals. Fifteen dorsal scale rows at mid-body. Two-hundred and one ventrals and 61/61 + 1 subcaudals, including a conical scale at the tail tip.

Colouration in alcohol of the head, neck and first dorsal scales (12 dorsal scales at midbody from between the parietal to end of pattern) is glossy black, including the upper and lower labials. The gulars are partially black with whitish background and the first ventrals are laterally black, while the middle ventral side is whitish. The ground colour of the dorsum is grey-yellowish-brown without maculation, while the ventral side is lighter, white.

*Variation.* Dentition features of the paratypes are similar to that of the holotype. Paratypes are similar to the holotype in most morphological characters (Table 2; Fig. 3). Exceptions include trapezoid shape of the internasals (HUJ.R21705; TAU.R15930; TAU.R17093); two postocular scales (HUJ.R21705; TAU.R15930); two post-temporal scales (HUJ.R21705, on the left side); the dorsal and ventral colour pattern are very similar to that of the holotype (BDS, 10–16).

*Habitat and ecology.* Specimens of *R. dayanae* **sp. nov.** were mostly found as road kill. The snake’s secretive lifestyle, its remote habitat and its presence, as far as we know, only in a strict nature reserve, hinder a full evaluation and detailed knowledge of its ecological preferences. Its biology, and that of the other *Rhynchocalamus* species, is poorly known. *Rhynchocalamus dayanae* **sp. nov.** is a reclusive, fossorial, ground-dwelling snake. It is mainly nocturnal, and has been observed active only at night on the ground and occasionally inside human households (TAU.R17093 was killed inside a house in the town of Mitzpe Ramon). Observations and collections of these snakes were carried out during the evenings and nights, at elevations between 700 and 1,000 m a.s.l. (G Vine, pers. comm., 2014). The species inhabits arid and rocky or stony steppes and sparsely vegetated areas, but is not found on sand (Fig. 6; Bar & Haimovitch, 2013; Werner, 2016). The diet consists predominantly of small insects such as ants, crickets and locusts (recorded for *R. melanocephalus*; Disi et al., 2001).

*Distribution. Rhynchocalamus dayanae* **sp. nov.** comprises to date only six specimens, collected from the Negev Mountain area in southern Israel, from the town of Mitzpe Ramon south-west up to the Egyptian border (Fig. 1). Most of the specimens of this newly-described species were observed and collected as road kills at the sides of main roads no. 40 (north-south direction) and no. 171 (from Mitzpe Ramon westwards to the Egyptian border). It probably also occurs on the Egyptian side of the border, but no specimens of *R. dayanae* **sp. nov.** from Egypt are known, although some specimens of *R. melanocephalus* are known from the Sinai Peninsula (Fig. 1; HUJ.R8856, HUJ.R8885, TAU.R12494); at the moment, therefore, it is considered endemic to Israel.

#### Conservation status

Not evaluated.

Proposal of common names:

English: Dayan’s Kukri Snake.

## Discussion

### Systematics of *Rhynchocalamus*

The snakes of the genus *Rhynchocalamus* are relatively rare, reclusive and poorly known. Consequently, the number of samples, observations and collected specimens is quite low, and natural history, life history, behavioural and ecological data are scarce. To date, only two studies have provided data on the systematics of the genus (Avci et al., 2015; Šmíd et al., 2015), revealing its sister phylogenetic relationship with *Lytorhynchus* within the Western Palearctic clade of Colubridae. These two studies, however, were each based only on four *Rhynchocalamus* specimens and on a single sequence of *Lytorhynchus diadema* (the type species of the genus *Lytorhynchus*). The broader sampling in our study, of both genera, strongly supports this phylogenetic hypothesis and their monophyly (as suggested by Šmíd et al., 2015; Fig. S1). Our results also support the phylogenetic separation between *Rhynchocalamus* and *Muhtarophis barani* (Avci et al., 2015), with the latter’s phylogenetic position still unresolved. Further interpretation of the phylogenetic relationships of *Rhynchocalamus* within the Western Palearctic clade is limited due to the low support values of the deepest nodes within the tree (see also Lawson et al., 2005; Vidal et al., 2007; Pyron, Burbrink & Wiens, 2013; Avci et al., 2015; Šmíd et al., 2015).

Our molecular results support *Rhynchocalamus* as a monophyletic, moderately diverse genus. The sampling of the only three previously known species of the genus revealed four distinct lineages. The genetic diversity among these four lineages is in concordance with the divergence documented among species within the Western Palearctic colubrid clade (e.g., Nagy et al., 2003; Nagy et al., 2004; Carranza et al., 2004), and is also supported by clear morphological differences (see Table 1; Figs. 4–5). These lineages represent four distinct species, but show incomplete lineage sorting in the *c-mos* nuclear network. The findings from this study provide support for the specific status of *R. arabicus*, *R. dayanae* **sp. nov.**, *R. melanocephalus*, and *R. satunini*. *Rhynchocalamus* species can be morphologically differentiated by several characteristics, such as the colour pattern of the body and the basic colour of the head (Figs. 4–5), and mensural variables (Table 1). Other traits consist of the number of ventral and subcaudal scales (higher number in *R. arabicus*), number of upper labials (six *vs*. seven in *R. satunini*), number of lower labials (eight *vs*. seven in *R. melanocephalus*) and number of postocular scales (one *vs*. two in *R. dayanae* **sp. nov.**).

The best-studied species of the genus, *R. melanocephalus*, was found to be paraphyletic (Fig. 2A). This species concealed an unidentified and morphologically similar arid species, described herein as *R. dayanae* **sp. nov.** This addition of a new species of snake to the fauna of Israel is surprising considering the long history of herpetological guides in the country (e.g., Haas, 1951; Barash & Hoofien, 1956; Arbel, 1984; Werner, 1988; Werner, 1995; Bouskila & Amitai, 2001; Bar & Haimovitch, 2013; Werner, 2016). This somewhat recent discovery may stem from the reclusive nature of these snakes, and because more specimens from southern Israel have been collected only recently. Subsequently, there are a mere six specimens known within the newly-discovered species in natural history collections, only four of which could be successfully used for genetic analyses (Tables S1 and S3). This new species from the Negev Mountain region in Israel is morphologically and genetically distinct, with a limited distribution from around the town of Mitzpe Ramon westward to the Egyptian border (Fig. 1). The specimens examined in this study reveal *R. dayanae* **sp. nov.** and *R. melanocephalus* to be allopatric species, separated by a distance of 15 km (between specimens HUJ.R22054, R22055 in locations 6–7 in Fig. 1 and HUJ.R21704, location 5). However, due to the low number of specimens from the area and of samples available, this assessment may change with the accumulation of more data. The molecular results reveal that *R. dayanae* **sp. nov.** is phylogenetically closely related to *R. arabicus* from Oman rather than to the geographically adjacent populations of *R. melanocephalus* in Israel northwards to Turkey. This relationship is surprising, as the two former species are located at a distance of about 2,500 km from one another (Fig. 1), whereas the closest collecting localities of *R. melanocephalus* and *R. dayanae* are just 15 km apart. As *Rhynchocalamus* snakes are rare and observations of them are scarce (specimens were not observed in several surveys in Arabia; e.g., Gasperetti, 1988; Schätti & Gasperetti, 1994; Schätti & Desvoignes, 1999; Van der Kooij, 2001; Gardner, 2013), it is plausible that a connection exist between populations, especially in desert habitats (as suggested in Gasperetti, 1988).

Specimens from the Sinai Peninsula (Table S3; HUJ.R8856; HUJ.R8885; TAU.R12494; Baha El Din, 2006) present a morphological combination of meristic characters similar both to *R. dayanae* **sp. nov.** (eight lower labials, 67%; four lower labials in contact with the anterior inframaxillars, 100%) and to *R. melanocephalus* (one post-ocular scale; two post-temporal scales; 13 dorsal and temporal scales surrounding the parietals). The Sinai specimens’ colouration is consistent with that of *R. melanocephalus* (gular scales and the upper and lower labials white). At this point, due to the low sample size, we cannot determine the taxonomic classification of the Sinai specimens and therefore cannot reject the presence of *R. dayanae* **sp. nov.** in the Sinai Peninsula. Because these specimens were kept for decades in formalin, the possibility of a genetic study was not possible.

The distribution of *R. melanocephalus* in Israel is not well defined, as it is mostly known from the Ecotone region in the northern Negev and northwards, and with small marginal population in the Negev desert (Arbel, 1984; Werner, 1988; Bouskila & Amitai, 2001; Bar & Haimovitch, 2013; Werner, 2016). An early Israeli reptile guide (Barash & Hoofien, 1956) noted that the southern desert populations of *R. melanocephalus* from the area of Sde Boker in the Negev displayed some morphological differences from the other, northern, specimens of the species, and may represent a new subspecies. We found that the black pattern of the head and the dorsal scales of the Negev populations in Israel (HUJ.R3653, HUJ.R8921, HUJ.R21833, R21834, HUJ.R22054, R22055, R22056, R22057, R22058; locations 6–12 in Fig. 1), adjacent to Sde Boker, do indeed differ from the Mediterranean populations and those from the desert areas in the Sinai Peninsula and Jordan. However, these differences are the sole indicators for differentiation, as their mensural and meristic traits are within the intraspecific variation of *R. melanocephalus* (Tables S1 and S3), and they are also genetically closely related (Fig. 2). An interesting finding arising from the phylogenetic analyses is that of the differentiation of the sole sample from Mt. Hermon in Israel (HUJ.R20967), which is otherwise morphologically very similar to other *R. melanocephalus* in terms of scalation and measurements. As no additional specimens from proximate localities in Lebanon or Syria are available, we are unable to account for this differentiation with the data at hand. In addition, sexual dimorphism is noted within *R. melanocephalus*. Females, in general, have a larger body size and a higher number of ventrals; males have relatively larger head dimensions and a higher count of sub-caudal scales (see also Werner, 2016).

The distinct lineage of *R. satunini* from Turkey and Iran, and the morphological revision, support its discrete specific status (as recommended by Reed & Marx, 1959; Baran, 1977; Avci et al., 2015; Šmíd et al., 2015). This molecular and morphological assessment contradicts Franzen & Bischoff (1995) contention that this species shows no morphological differences from *R. melanocephalus*. That said, the lack of sufficient sampling in Iran and the southern Caucasus, along with the species’ disjunct distribution (Fig. 1 and Fig. S4), and the relatively high genetic diversity within the species, suggest that additional lineages may well be awaiting description.

The enigmatic *R. arabicus*, one of the rarest snakes in the world, represents its own lineage, based on the sole sample available for the phylogenetic analyses. This snake is known only from one specimen (the holotype) as the recently sampled individual was not collected (Šmíd et al., 2015). Ecological data, such as habitat preferences or biology, are also lacking for this snake. The newly-sampled specimen from Oman was observed during the night, resting between stones in a vegetated wadi with permanent water (Šmíd et al., 2015). This observation accords with the nocturnal behaviour of other *Rhynchocalamus* species.

### Biogeographic assessment of *Rhynchocalamus*

The estimated divergence times in this study present the first calibrated phylogeny of *Rhynchocalamus* to date. The estimated times indicate a very old, Late-Oligocene, divergence between *Lytorhynchus* and *Rhynchocalamus* at ca. 26 Mya (Fig. 2A). *Lytorhynchus* is comprised of six species (Uetz & Hošek, 2016) and is mostly distributed in south-western Asia with a single species also found in Africa (*i*.*e*. *L. diadema*; Sindaco, Venchi & Grieco, 2013). *Lytorhynchus* is sympatric with *Rhynchocalamus* in south-western Iran and the Middle East, though they predominantly occur in different habitats (i.e., *Lytorhynchus* is a sand dweller whereas *Rhynchocalamus* occurs on heavier soils; Khan, 1996; Disi et al., 2001; Bar & Haimovitch, 2013). The close phylogenetic relationship between the two mostly south-west Asian *Lytorhynchus* and *Rhynchocalamus* may help to elucidate the biogeography of the latter genus.

The Late-Oligocene divergence between *Lytorhynchus* and *Rhynchocalamus* may have resulted from the changing landscapes and habitat fragmentation around the contact region of the Paratethys and Mediterranean seas with the Indian Ocean in south-western Asia (i.e., today’s south-eastern Turkey and northern Syria). This region is where the collision of the African-Arabian plate with the Eurasian landmass took place during the Early Miocene, 16–18 Mya (Rögl, 1999; Harzhauser & Piller, 2007). The landscape of this region underwent continual changes during the Oligocene and Miocene due to several environmental changes, such as the tectonic motions of the Arabian and African plates resulting in the closure or opening of marine seaways, sea level fluctuations, the uplift of mountain ridges and the creation of regional fault systems (Rögl, 1999; Griffin, 2002; Harzhauser & Piller, 2007; Inwood et al., 2009). The presence of two Levantine species, *R. melanocephalus* and *R. dayanae* **sp. nov.**, and another in Arabia, *R. arabicus*, may allude to the ancestral range of *Rhynchocalamus* in the Levant (Fig. 1).

Radiation within *Rhynchocalamus* began with the separation of the northern *R. satunini* from Iran and Turkey during the Middle Miocene, approximately 15 Mya (Fig. 2A). This diversification is likely to have been the result of a vicariance event resulting from the severing of the *Gomphotherium* land bridge in south-eastern Turkey, as was also suggested for other reptilian taxa such as the lacertid genus *Apathya* (Kapli et al., 2013). This Levantine land bridge, resulting from the African-Arabian-Eurasian tectonic connection, linked Africa and Eurasia ca. 16–18 Mya (Rögl, 1999; Harzhauser & Piller, 2007; Inwood et al., 2009). The *Gomphotherium* land bridge had major biogeographical implications, enabling faunal dispersal from Africa to Eurasia and *vice versa* (Tchernov, 1992; Wüster et al., 2007; Harzhauser et al., 2007; Pook et al., 2009; Zhou et al., 2012). The marine connection between the Mediterranean and Paratethys seas to the Indian Ocean, severing this land bridge, is hypothesized to have been re-established in the Middle Miocene, around 15–16 Mya (Rögl, 1999; Popov et al., 2004; Harzhauser & Piller, 2007). The re-opening of the marine connection and the disconnection of Eurasia from Africa-Arabia may have been responsible for the separation of the northern *R. satunini* from the ancestral, southern, population of the other *Rhynchocalamus* taxa.

The Middle Miocene diversification of *R. arabicus, R. dayanae* **sp. nov.** and *R. melanocephalus* is difficult to interpret due to the paucity of known localities and samples in the Arabian Peninsula connecting *R. arabicus* to the other two species. The divergence between *R. arabicus* and *R. dayanae* **sp. nov.**, ca. 9 Mya, may be related to several environmental events in the Arabian region. It has been hypothesized that continuous Middle-Late Miocene tectonic motions of the Arabian plate caused geological instability in north-western Arabia, leading to the creation of the Aqaba-Levant transform and periodic volcanic activity (Bosworth, Huchon & McClay, 2005). In addition, a temporal land connection of halite deposits existed between Africa and Arabia in the Red Sea, which later became submerged with the expansion and refill of the Red Sea (∼14–10 Mya; Richardson & Arthur, 1988; Rögl, 1999; Bosworth, Huchon & McClay, 2005). These events might have caused habitat fragmentation and facilitated radiation within *Rhynchocalamus* in the southern Levant and Arabia. The Middle Miocene global climate change resulted in an aridification process of the mid-latitude continental regions, triggering the expansion of sand areas in Arabia and North Africa (Flower & Kennett, 1994; Le Houérou, 1997; Edgell, 2006). The formation and progression of sandy habitats probably promoted vicariance and/or isolation of hard-substrate specific taxa, such as *Rhynchocalamus*, restricting the snakes to their current distribution, similar to what has been suggested for other reptiles, such as the geckos of the genus *Stenodactylus* (Metallinou et al., 2012), the Arid clade of *Hemidactylus* (Carranza & Arnold, 2012; Šmíd et al., 2013), the *Ptyodactylus hasselquistii* species complex (Metallinou et al., 2015), the lacertid genus *Acanthodactylus* (Tamar et al., 2016a) and the agamid genus *Pseudotrapelus* (Tamar et al., 2016b). The sands of the north-west Negev in Israel, extending westwards in the northern Sinai Peninsula, restrict the distribution of *R. melanocephalus* and *R. dayanae* **sp. nov.** in Israel and Egypt (Baha El Din, 2006; Bar & Haimovitch, 2013). The potential distribution of *R. dayanae* **sp. nov.** and the limited range of *R. melanocephalus* in southern Israel suggests strict habitat preferences (Fig. S4), indicating that soil type and temperature seasonality, respectively, play an important role in explaining the potential distribution of these species. The sand deserts in the Arabian Peninsula, such as An Nafud, Rub’ al Khali and Sharqiyah, probably restrict *Rhynchocalamus* snakes to the mountainous coastal areas of the peninsula. Thus it may be hypothesized that the distribution of these snakes are limited to these areas, though additional samples and specimens from this range are crucial in order to enable further assessment.

The discovery of *R. dayanae* **sp. nov.** and its restricted range within Israel highlight the need for an IUCN Red List assessment to determine its conservation status. In light of the separation of the new species, *R. melanocephalus*, previously assessed as Least Concern (Amr et al., 2012), should also be reassessed.

## Supplemental Information

10.7717/peerj.2769/supp-1 Table S1 Data on the genetic datasets and the genetic distance within *Rhynchocalamus* Taxon names correspond to changes proposed in this paper. (A) *Rhynchocalamus* dataset. Localities for *Rhynchocalamus* are presented in Fig. 1. (*) Specimen used for the morphological examinations; (i) Haplotypes used for the species delimitation analyses (*n* = 28); (ii) Representatives used for the divergence time estimation (*n* = 6); (B) dataset of the Western Palearctic colubrid clade, including new sequences of *Lytorhynchus* and *Muhtarophis barani*; (C) Pairwise uncorrected genetic divergence (*p*-distance) between the *Rhynchocalamus* taxa (*12S*/*16S*; below the diagonal) and *cytb* (above the diagonal), and within each taxa (*12S*/*16S*/*cytb*).

10.7717/peerj.2769/supp-2 Table S2 Information on the phylogenetic analyses (A) Species-set A; (B) species-set B; (C) species-set C.

10.7717/peerj.2769/supp-3 Table S3 Morphological dataset of all examined specimens of *Rhynchocalamus* Taxon names correspond to changes proposed in this paper. Specimens used in the genetic analyses (genetic; *n* = 26) are presented in Fig. 1. (*) Specimens of *R. melanocephalus* from the Negev region in Israel (*n* = 9). Dataset of the mensural characters is presented in millimetres. Meristic characters are presented with the left/right sides. Character and museum abbreviations are listed in the Material and Methods section.

10.7717/peerj.2769/supp-4 Table S4 Statistical results of the morphological dataset *P*-values in bold indicate high level of statistical significance. Abbreviations of each morphological variable, as in Table S3, are listed in the Material and Methods section.

10.7717/peerj.2769/supp-5 Figure S1 Maximum likelihood gene tree of the Western Palearctic colubrid clade The tree was inferred from the concatenated dataset of the mitochondrial (*12S, 16S, cytb*) and nuclear (*c-mos*) gene fragments (dataset 1). Support values near the nodes indicate ML bootstrap and BI posterior probability (values ≥ 70%, ≥ 0.95; ML, BI, respectively). Sample codes and colours correlate to specimens in Table S1 and in Figs. 1–2,Tables S2–S4. Taxon names correspond to changes proposed in this paper.

10.7717/peerj.2769/supp-6 Figure S2 Results of the mPTP species delimitation analysis The Analysis was based on haplotype mtDNA (dataset 4). Asterisk indicates representatives used for the divergence time estimation analysis. Sample codes correlate to specimens in Table S1 and in Figs. 1–2,Tables S1–S3.

10.7717/peerj.2769/supp-7 Figure S3 Time calibrated Bayesian Inference tree of the Western Palearctic Colubrine clade The tree was inferred from the concatenated dataset of the mitochondrial (*12S, 16S, cytb*) and nuclear (*c-mos*) gene fragments (dataset 1), based on two calibration points of *Hemorrhois* and *Hierophis* subgroups (blue circles; see Material and Methods; dataset 2). Median age estimates are provided near the nodes with bars representing the 95% highest posterior densities (HPD). Black circles represent nodes with high support (posterior probability values ≥ 0.95). Sample codes correlate to specimens in Table S1 and in Figs. 1–2,Tables S1–S2.

10.7717/peerj.2769/supp-8 Figure S4 Species distribution models of *Rhynchocalamus* taxa Projected Maxent models of the potential presence/absence of *R. dayanae* **sp. nov.**, *R. melanocephalus* and *R. satunini*. The cut-off threshold was Maximum Training Sensitivity plus Specificity (MTSS) to convert the continuous Maxent output to binary maps. Colours correspond to taxa in Table S1 and in Figs. 1–2, Figs. S1–S2.

10.7717/peerj.2769/supp-9 Supplemental Information 1 For review only

10.7717/peerj.2769/supp-10 Supplemental Information 2 For review only

10.7717/peerj.2769/supp-11 Supplemental Information 3 For review only

10.7717/peerj.2769/supp-12 Supplemental Information 4 For review only

## Additional Information and Declarations

### Field Study Permissions

The following information was supplied relating to field study approvals (i.e., approving body and any reference numbers): Collecting permit for this study in Turkey was issued by the Ministry of Forestry and Water Affairs from the Republic of Turkey (2014#51946).

### DNA Deposition

The following information was supplied regarding the deposition of DNA sequences: All sequences have been deposited in GenBank database. Accession numbers for the DNA sequences included in this study are provided in Table S1.

### Data Availability

The following information was supplied regarding data availability: All molecular or morphological data related to this manuscript are available in Tables S1 and S3, respectively.

### New Species Registration

The following information was supplied regarding the registration of a newly described species: *Rhynchocalamus* dayanae urn:lsid:zoobank.org:act:8FC77FE5-1262-4631-AC2D-F202B5A9003C Publication LSID: urn:lsid:zoobank.org:pub:1E72A585-C0C3-4835-9466-25E8A7C9FADB.

## References

[ref-1] Amr ZS, Disi AM (2011). Systematics, distribution and ecology of the snakes of Jordan. Vertebrate Zoology.

[ref-2] Amr ZS, Egan DM, Nilson G, Kumlutaş Y, Baha El Din S, Sadek R, Lymberakis P, Ugurtas IH, Werner YL, Tok V, Sevinç M, Hraoui-Bloquet S, Crochet PA, Kaska Y, Avci A (2012). *Rhynchocalamus melanocephalus*. The IUCN red list of threatened species 2012.

[ref-3] Ananjeva NB, Orlov NL, Khalikov RG, Darevsky IS, Ryabov IS, Barabanov AV (2006). The Reptiles of North Eurasia. Taxonomic diversity, distribution, conservation status [this comprises the territory of the former Soviet Union and Mongolia].

[ref-4] Araújo MB, Pearson RG, Thuiller W, Erhard M (2005). Validation of species-climate impact models under climate change. Global Change Biology.

[ref-5] Arbel A (1984). Reptiles and amphibians. Encyclopedia of plants and animals of the land of Israel.

[ref-6] Avci A, Dinçaslan YE, Ilgaz Ç, Üzüm N (2008). Contributions to the distribution and morphology of *Rhynchocalamus melanocephalus melanocephalus* (Jan, 1862) (Reptilia, Colubridae) in Turkey. North-Western Journal of Zoology.

[ref-7] Avci A, Ilgaz Ç, Kumlutas Y, Olgun K, Baran I (2007). Morphology and distribution of *Rhynchocalamus melanocephalus* (NIKOLSKY, 1899) in Turkey. Herpetozoa.

[ref-8] Avci A, Ilgaz Ç, Rajabizadeh M, Yilmaz C, Üzüm N, Adriaens D, Kumlutaş Y, Olgun K (2015). Molecular phylogeny and micro-CT scanning revealed extreme cryptic biodiversity in Kukri snake, *Muhtarophis* gen. nov., a new genus for *Rhynchocalamus barani* (Serpentes: Colubridae). Russian Journal of Herpetology.

[ref-9] Baha El Din SM (1994). A contribution to the herpetology of Sinai. British Herpetological Society Bulletin.

[ref-10] Baha El Din SM (2006). A guide to reptiles & amphibians of Egypt.

[ref-11] Bandelt HJ, Forster P, Röhl A (1999). Median-joining networks for inferring intraspecific phylogenies. Molecular Biology and Evolution.

[ref-12] Bar A, Haimovitch G (2011). A field guide to reptiles and amphibians of Israel.

[ref-13] Bar A, Haimovitch G (2013). A field guide to reptiles and amphibians of Israel.

[ref-14] Baran I (1977). Some rare species of snakes from Turkey. Annalen Des Naturhistorischen Museums in Wien.

[ref-15] Barash A, Hoofien JH (1956). Reptiles of Israel.

[ref-16] Blaxter ML (2004). The promise of DNA taxonomy. Philosophical Transactions of the Royal Society of London B.

[ref-17] Bodenheimer FS (1944). Introduction into the knowledge of the Amphibia and Reptilia of Turkey. Reviews by Faculty of Sciences, University of Istanbul, Series B.

[ref-18] Bosworth W, Huchon P, McClay K (2005). The Red Sea and Gulf of Aden basins. Journal of African Earth Sciences.

[ref-19] Boulenger GA (1894). Second report on additions to the lizard collection in the natural history museum. Proceedings of the Scientific Meetings of the Zoological Society.

[ref-20] Bouskila A, Amitai P (2001). Handbook of amphibians & reptiles of Israel.

[ref-21] Carranza S, Arnold EN (2012). A review of the geckos of the genus *Hemidactylus* (Squamata: Gekkonidae) from Oman based on morphology, mitochondrial and nuclear data, with descriptions of eight new species. Zootaxa.

[ref-22] Carranza S, Arnold EN, Wade E, Fahd S (2004). Phylogeography of the false smooth snakes, *Macroprotodon* (Serpentes, Colubridae): mitochondrial DNA sequences show European populations arrived recently from Northwest Africa. Molecular Phylogenetics and Evolution.

[ref-23] Castresana J (2000). Selection of conserved blocks from multiple alignments for their use in phylogenetic analysis. Molecular Biology and Evolution.

[ref-24] Chernov SA (1937). Field guide of snakes, lizards and tortoises of Armenia.

[ref-25] Darevsky I (1970). Systematic status of *Rhynchocalamus melanocephalus satunini* Nik. (Serpentes, Colubridae) previously included in the genus *Oligodon*. Zoologicheskii Zhurnal.

[ref-26] Dayrat B (2005). Towards integrative taxonomy. Biological Journal of the Linnean Society.

[ref-27] Disi AM, Modry D, Necas P, Rifai L (2001). Amphibians and reptiles of the Hashemite Kingdom of Jordan: an atlas and field guide.

[ref-28] Drummond AJ, Suchard MA, Xie D, Rambaut A (2012). Bayesian phylogenetics with BEAUti and the BEAST 1.7. Molecular Biology and Evolution.

[ref-29] Edgell HS (2006). Arabian deserts: nature, origin and evolution.

[ref-30] Felsenstein J (1985). Confidence limits on phylogenies: an approach using the bootstrap. Evolution.

[ref-31] Flot JF (2010). SeqPHASE: a web tool for interconverting PHASE input/output files and FASTA sequence alignments. Molecular Ecology Resources.

[ref-32] Flower BP, Kennett JP (1994). The middle Miocene climatic transition: east Antarctic ice sheet development, deep ocean circulation and global carbon cycling. Palaeogeography, Palaeoclimatology, Palaeoecology.

[ref-33] Franzen M, Bischoff W (1995). Erstnachweis von *Rhynchocalamus melanocephalus melanocephalus* für die Turkei. Salamandra.

[ref-34] Gardner AS (2013). The amphibians and reptiles of Oman and the UAE.

[ref-35] Gasperetti J (1988). Snakes of Arabia. Fauna of Saudi Arabia.

[ref-36] Griffin DL (2002). Aridity and humidity: two aspects of the late Miocene climate of North Africa and the Mediterranean. Palaeogeography, Palaeoclimatology, Palaeoecology.

[ref-37] Günther A (1864). Report on a collection of reptiles and fishes from Palestine. Proceedings of the Scientific Meetings of the Zoological Society.

[ref-38] Haas G (1951). On the present state of our knowledge of the herpetofauna of Palestine. Bulletin of the Research Council of Israel.

[ref-39] Haas G (1952). Two collections of reptiles from Iraq with descriptions of two new forms. Copeia.

[ref-40] Harzhauser M, Kroh A, Mandic O, Piller WE, Göhlich U, Reuter M, Berning B (2007). Biogeographic responses to geodynamics: a key study all around the Oligo-Miocene Tethyan Seaway. Zoologischer Anzeiger-A Journal of Comparative Zoology.

[ref-41] Harzhauser M, Piller WE (2007). Benchmark data of a changing sea–palaeogeography, palaeobiogeography and events in the Central Paratethys during the Miocene. Palaeogeography, Palaeoclimatology, Palaeoecology.

[ref-42] Hebert PDN, Cywinska A, Ball SL, DeWaard JR (2003). Biological identifications through DNA barcodes. Proceedings of the Royal Society of London B: Biological Sciences.

[ref-43] Hijmans RJ, Cameron SE, Parra JL, Jones PG, Jarvis A (2005). Very high resolution interpolated climate surfaces for global land areas. International Journal of Climatology.

[ref-44] Huelsenbeck JP, Rannala B (2004). Frequentist properties of Bayesian posterior probabilities of phylogenetic trees under simple and complex substitution models. Systematic Biology.

[ref-45] Inwood J, Anderson MW, Morris A, Robertson AH (2009). Successive structural events in the Hatay ophiolite of southeast Turkey: distinguishing oceanic, emplacement and post-emplacement phases of faulting. Tectonophysics.

[ref-46] Ivanov M (2002). The oldest known Miocene snake fauna from Central Europe: Merkur−North locality, Czech Republic. Acta Palaeontologica Polonica.

[ref-47] Jan G (1862). Enumerazione sistematica delle specie d’Ofidi del gruppo Calamaridae. Archivio per la Zoologia, l’Anatomia e la Fisiologia G. Canestrini.

[ref-48] Kapli P, Botoni D, Ilgaz Ç, Kumlutaş Y, Avcı A, Rastegar-Pouyani N, Fathinia B, Lymberakis P, Ahmadzadeh F, Poulakakis N (2013). Molecular phylogeny and historical biogeography of the Anatolian lizard *Apathya* (Squamata, Lacertidae). Molecular Phylogenetics and Evolution.

[ref-49] Kapli P, Lutteropp S, Zhang J, Kobert K, Pavlidis P, Stamatakis A, Flouri T (2016). Multi-rate Poisson tree processes for single-locus species delimitation under maximum likelihood and Markov chain Monte Carlo. bioRxiv.

[ref-50] Katoh K, Standley DM (2013). MAFFT multiple sequence alignment software version 7: improvements in performance and usability. Molecular Biology and Evolution.

[ref-51] Khan MS (2006). Amphibians and reptiles of Pakistan.

[ref-52] Kocher TD, Thomas WK, Meyer A, Edwards SV, Pääbo S, Villablanca FX, Wilson AC (1989). Dynamics of mitochondrial DNA evolution in animals: amplification and sequencing with conserved primers. Proceedings of the National Academy of Sciences of the United States of America.

[ref-53] Kornilios P, Giokas S, Lymberakis P, Sindaco R (2013). Phylogenetic position, origin and biogeography of Palearctic and Socotran blind-snakes (Serpentes: Typhlopidae). Molecular Phylogenetics and Evolution.

[ref-54] Kumar S, Stecher G, Tamura K (2016). MEGA7: molecular evolutionary genetics analysis version 7.0 for bigger datasets. Molecular Biology and Evolution.

[ref-55] Lanfear R, Calcott B, Ho SYW, Guindon S (2012). PartitionFinder: combined selection of partitioning schemes and substitution models for phylogenetic analyses. Molecular Biology and Evolution.

[ref-56] Lawson R, Slowinski JB, Crother BI, Burbrink FT (2005). Phylogeny of the Colubroidea (Serpentes): new evidence from mitochondrial and nuclear genes. Molecular Phylogenetics and Evolution.

[ref-57] Le Houérou HN (1997). Climate, flora and fauna changes in the Sahara over the past 500 million years. Journal of Arid Environments.

[ref-58] Lenk P, Kalyabina S, Wink M, Joger U (2001). Evolutionary relationships among the true vipers (Reptilia: Viperidae) inferred from mitochondrial DNA sequences. Molecular Phylogenetics and Evolution.

[ref-59] Metallinou M, Arnold EN, Crochet PA, Geniez P, Brito JC, Lymberakis P, Baha El Din S, Sindaco R, Robinson M, Carranza S (2012). Conquering the Sahara and Arabian deserts: systematics and biogeography of *Stenodactylus* geckos (Reptilia: Gekkonidae). BMC Evolutionary Biology.

[ref-60] Metallinou M, Červenka J, Crochet PA, Kratochvíl L, Wilms T, Geniez P, Shobrak MY, Brito JC, Carranza S (2015). Species on the rocks: systematics and biogeography of the rock-dwelling *Ptyodactylus* geckos (Squamata: Phyllodactylidae) in North Africa and Arabia. Molecular Phylogenetics and Evolution.

[ref-61] Miller M, Pfeiffer W, Schwartz T (2010). Creating the CIPRES science gateway for inference of large phylogenetic trees.

[ref-62] Nagy ZT, Joger U, Wink M, Glaw F, Vences M (2003). Multiple colonization of Madagascar and Socotra by colubrid snakes: evidence from nuclear and mitochondrial gene phylogenies. Proceedings of the Royal Society of London B.

[ref-63] Nagy ZT, Lawson R, Joger U, Wink M (2004). Molecular systematics of racers, whipsnakes and relatives (Reptilia: Colubridae) using mitochondrial and nuclear markers. Journal of Zoological Systematics and Evolutionary Research.

[ref-64] Nikolsky AM (1899). *Contia statunini* n. sp., and *Agama ruderata* Oliv., from Transcaucasia. Annuaire Musée Zoologique de l’Académie Impériale des Sciences de St.-Pétersbourg.

[ref-65] Olgun K, Avci A, Ilgaz C, Üzüm N, Yilmaz C (2007). A new species of *Rhynchocalamus* (Reptilia: Serpentes: Colubridae) from Turkey. Zootaxa.

[ref-66] Padial JM, Miralles A, De la Riva I, Vences M (2010). Review: the integrative future of taxonomy. Frontiers in Zoology.

[ref-67] Palumbi S, Hillis DM, Moritz C, Mable BK (1996). Nucleic acids II: the polymerase chain reaction. Molecular systematics.

[ref-68] Phillips SJ, Anderson RP, Schapire RE (2006). Maximum entropy modeling of species geographic distributions. Ecological Modelling.

[ref-69] Phillips SJ, Dudík M (2008). Modeling of species distributions with Maxent: new extensions and a comprehensive evaluation. Ecography.

[ref-70] Pook CE, Joger U, Stümpel N, Wüster W (2009). When continents collide: phylogeny, historical biogeography and systematics of the medically important viper genus *Echis* (Squamata: Serpentes: Viperidae). Molecular Phylogenetics and Evolution.

[ref-71] Popov SV, Rögl F, Rozanov AY, Steininger FF, Shcherba IG, Kovac M (2004). Lithological-paleogeographic maps of Paratethys 10 maps Late Eocene to Pliocene. Courier Forschungsinstitut Senckenberg.

[ref-72] Pyron R, Burbrink F, Wiens J (2013). A phylogeny and revised classification of Squamata, including 4,161 species of lizards and snakes. BMC Evolutionary Biology.

[ref-73] Rambaut A, Suchard MA, Xie D, Drummond AJ (2014). Tracer v1.6. http://beast.bio.ed.ac.uk/Tracer.

[ref-74] Reed CA, Marx H (1959). A herpetological collection from northeastern Iraq. Transactions of the Kansas Academy of Science.

[ref-75] Richardson M, Arthur MA (1988). The Gulf of Suez—northern Red Sea neogene rift: a quantitive basin analysis. Marine and Petroleum Geology.

[ref-76] Rögl F (1999). Mediterranean and paratethys. Facts and hypotheses of an oligocene to miocene paleogeography (short overview). Geologica Carpathica.

[ref-77] Schatti B, Desvoignes A (1999). The herpetofauna of southern Yemen and the Sokotra Archipelago.

[ref-78] Schätti B, Gasperetti J (1994). A contribution to the herpetofauna of Southwest Arabia. Fauna of Saudi Arabia.

[ref-79] Schmidt KP (1933). A new snake (*Rhynchocalamus arabicus*) from Arabia. Zoological Series of Field Museum of Natural History.

[ref-80] Shimodaira H (2002). An approximately unbiased test of phylogenetic tree selection. Systematic Biology.

[ref-81] Shimodaira H, Hasegawa M (1999). Multiple comparisons of log-likelihoods with applications to phylogenetic inference. Molecular Biology and Evolution.

[ref-82] Shimodaira H, Hasegawa M (2001). CONSEL: for assessing the confidence of phylogenetic tree selection. Bioinformatics.

[ref-83] Silvestro D, Michalak I (2012). RaxmlGUI: a graphical front-end for RAxML. Organisms Diversity and Evolution.

[ref-84] Sindaco R, Venchi A, Grieco C (2013). The reptiles of the western Palearctic. 2. Annotated checklist and distributional atlas of the snakes of Europe, North Africa, Middle East and Central Asia, with an update to the Vol. 1.

[ref-85] Sites JW, Marshall JC (2003). Delimiting species: a renaissance issue in systematic biology. Trends in Ecology & Evolution.

[ref-86] Šmíd J, Carranza S, Kratochvíl L, Gvoždík V, Nasher AK, Moravec J (2013). Out of Arabia: a complex biogeographic history of multiple vicariance and dispersal events in the gecko genus *Hemidactylus* (Reptilia: Gekkonidae). PLoS ONE.

[ref-87] Šmíd J, Martínez G, Gebhart J, Aznar J, Gállego J, Göçmen B, De Pous P, Tamar K, Carranza S (2015). Phylogeny of the genus *Rhynchocalamus* (Reptilia; Colubridae) with a first record from the Sultanate of Oman. Zootaxa.

[ref-88] Stamatakis A (2006). RAxML-VI-HPC: maximum likelihood-based phylogenetic analyses with thousands of taxa and mixed models. Bioinformatics.

[ref-89] Stephens M, Scheet P (2005). Accounting for decay of linkage disequilibrium in haplotype inference and missing-data imputation. The American Journal of Human Genetics.

[ref-90] Stephens M, Smith NJ, Donnelly P (2001). A new statistical method for haplotype reconstruction from population data. The American Journal of Human Genetics.

[ref-91] Stümpel N, Joger U (2009). Recent advances in phylogeny and taxonomy of near and Middle Eastern Vipers–an update. ZooKeys.

[ref-92] Talavera G, Castresana J (2007). Improvement of phylogenies after removing divergent and ambiguously aligned blocks from protein sequence alignments. Systematic Biology.

[ref-93] Tamar K, Carranza S, Sindaco R, Moravec J, Trape JF, Meiri S (2016a). Out of Africa: phylogeny and biogeography of the widespread genus *Acanthodactylus* (Reptilia: Lacertidae). Molecular Phylogenetics and Evolution.

[ref-94] Tamar K, Scholz S, Crochet PA, Geniez P, Meiri S, Schmitz A, Wilms A, Carranza S (2016b). Evolution around the red sea: systematics and biogeography of the agamid genus *Pseudotrapelus* (Squamata: Agamidae) from North Africa and Arabia. Molecular Phylogenetics and Evolution.

[ref-95] Tautz D, Arctander P, Minelli A, Thomas RH, Vogler AP (2003). A plea for DNA Taxonomy. Trends in Ecology and Evolution.

[ref-96] Tchernov E (1992). The Afro-Arabian component in the Levantine mammalian fauna-a short biogeographical review. Israel Journal of Zoology.

[ref-97] Tonini JFR, Beard KH, Ferreira RB, Jetz W, Pyron RA (2016). Fully-sampled phylogenies of squamates reveal evolutionary patterns in threat status. Biological Conservation.

[ref-98] Uetz P, Hošek J (2016). The reptile database. http://www.reptile-database.org.

[ref-99] Utiger U, Helfenberger N, Schätti B, Schmidt C, Ruf M, Ziswiler V (2002). Molecular systematics and phylogeny of old and new world ratsnakes, *Elaphe* Auct., and related genera (Reptilia, Squamata, Colubridae). Russian Journal of Herpetology.

[ref-100] Van der Kooij J (2001). The herpetofauna of the Sultanate of Oman: part 4: the terrestrial snakes. Podarcis.

[ref-101] Vidal N, Delmas AS, David P, Cruaud C, Couloux A, Hedges SB (2007). The phylogeny and classification of caenophidian snakes inferred from seven nuclear protein-coding genes. Comptes Rendus Biologies.

[ref-102] Vogler AP, Monaghan MT (2007). Recent advances in DNA taxonomy. Journal of Zoological Systematics and Evolutionary Research.

[ref-103] Wallach V, Williams KL, Boundy J (2014). Snakes of the world. A catalogue of living and extinct species.

[ref-104] Warren DL, Glor RE, Turelli M (2010). ENMTools: a toolbox for comparative studies of environmental niche models. Ecography.

[ref-105] Werner F (1905). Einige für Kleinasien neue Reptilien. Zoologischer Anzeiger, Leipzig.

[ref-106] Werner YL, Yom-Tov Y, Tchernov E (1988). Herpetofaunal survey of Israel (1950–1985), with comments on Sinai and Jordan and on zoogeographical heterogeneity. The zoogeography of Israel. The distribution and abundance at a zoogeographical crossroad.

[ref-107] Werner YL (1995). A guide to the reptiles and amphibians of Israel.

[ref-108] Werner YL (2016). Reptile life in the land of Israel, with comments on adjacent regions.

[ref-109] Wilcox TP, Zwickl DJ, Heath TA, Hillis DM (2002). Phylogenetic relationships of the dwarf boas and a comparison of Bayesian and bootstrap measures of phylogenetic support. Molecular Phylogenetics and Evolution.

[ref-110] Wüster W, Crookes S, Ineich I, Mané Y, Pook CE, Trape JF, Broadley DG (2007). The phylogeny of cobras inferred from mitochondrial DNA sequences: evolution of venom spitting and the phylogeography of the African spitting cobras (Serpentes: Elapidae: *Naja nigricollis* complex). Molecular Phylogenetics and Evolution.

[ref-111] Wüster W, Peppin L, Pook CE, Walker DE (2008). A nesting of vipers: phylogeny and historical biogeography of the Viperidae (Squamata: Serpentes). Molecular Phylogenetics and Evolution.

[ref-112] Zhang J, Kapli P, Pavlidis P, Stamatakis A (2013). A general species delimitation method with applications to phylogenetic placements. Bioinformatics.

[ref-113] Zhou L, Su YCF, Thomas DC, Saunders BMK (2012). ‘Out-of-Africa’ dispersal of tropical floras during the Miocene climatic optimum: evidence from Uvaria (Annonaceae). Journal of Biogeography.

