# Supplementary material for: An integrative systematic revision and biogeography of Rhynchocalamus snakes (Reptilia, Colubridae) with a description of a new species from Israel
Source: PeerJ. 2016 Dec 22;4:e2769. doi: 10.7717/peerj.2769 (PMC5183090; doi:10.7717/peerj.2769)
Supplement: Table S1 — Taxon names correspond to changes proposed in this paper. (A) Rhynchocalamus dataset. Localities for Rhynchocalamus are presented in Fig. 1. (*) Specimen used for the morphological examinations; (i) Haplotypes used for the species delimitation analyses (n = 28); (ii) Representatives used for the divergence time estimation (n = 6); (B) dataset of the Western Palearctic colubrid clade, including new sequences of Lytorhynchus and Muhtarophis barani; (C) Pairwise uncorrected genetic divergence (p-distance) between the Rhynchocalamus taxa (12S/16S; below the diagonal) and cytb (above the diagonal), and within each taxa (12S/16S/cytb). [file peerj-04-2769-s001.docx]

(A)

| **Locality** | **Species** | **Specimen code^1^** | ***12S*** | ***16S*** | ***cytb*** | **c-*mos*** | **Country** | **Locality** | **Latitude** | **Longitude** | |
| --- | --- | --- | --- | --- | --- | --- | --- | --- | --- | --- | --- |
| 1 (i,ii) * | *R. arabicus* | CN4780 | KT878842 | KT878847 | KT878854 | KT878851 | Oman | Wadi Ayun, Dhofar | 17.252 | | 53.887 |
| 2 (i,ii) * | *R. dayanae* **sp. nov.** | TAU.R15930 | KX909282 | KX909314 | KX909331 | KX909360 | Israel | Road no. 10, Nahal Batur | 30.392 | | 34.591 |
| 3 (i) * | *R. dayanae* **sp. nov.** | TAU.R17093 | KX909283 | KX909313 | KX909332 | KX909361 | Israel | Mitzpe Ramon | 30.610 | | 34.802 |
| 4 (i) * | *R. dayanae* **sp. nov.** | HUJ.R21705 | KX909281 | KX909315 | KX909330 | - | Israel | Road no. 171, between Borot Lotz and Harif Mt. | 30.501 | | 34.588 |
| 5 (i) * | *R. dayanae* **sp. nov.** | HUJ.R21704 | KX909280 | KX909312 | KX909329 | - | Israel | Road no. 40 near Nafha prison | 30.731 | | 34.770 |
| 6 (i,ii) * | *R. melanocephalus* | HUJ.R22054 | KX909266 | KX909299 | KX909333 | KX909356 | Israel | Road no. 40, between Qibbuz & school of Sde Boqer | 30.864 | | 34.777 |
| 7 (i) * | *R. melanocephalus* | HUJ.R22055 | KX909267 | KX909300 | KX909337 | KX909357 | Israel | Road no. 40, between Qibbuz & school of Sde Boqer | 30.864 | | 34.777 |
| 8 (i) * | *R. melanocephalus* | HUJ.R22058 | KX909270 | KX909303 | - | KX909358 | Israel | Road no. 204, between Haluqim junction and Merhav-Am | 30.889 | | 34.811 |
| 9 (i) * | *R. melanocephalus* | HUJ.R21833 | KX909265 | - | - | - | Israel | Exit from the big crater towards Yeruham | 30.972 | | 34.970 |
| 10 (i) * | *R. melanocephalus* | HUJ.R21834 | KX909271 | - | - | - | Israel | The big crater (Makhtesh Gadol) | 30.974 | | 34.966 |
| 11 (i) * | *R. melanocephalus* | HUJ.R22056 | KX909268 | KX909301 | KX909336 | KX909359 | Israel | Ascent from the big crater to Yeruham | 30.969 | | 34.976 |
| 12 * | *R. melanocephalus* | HUJ.R22057 | KX909269 | KX909302 | KX909334 | KX909346 | Israel | Ashalim (turn from road to "Ru'ah Midbar" farm) | 30.976 | | 34.737 |
| 13 (i) | *R. melanocephalus* | HUJ.R22241 | KX909272 | KX909304 | KX909335 | KX909347 | Israel | Ramat Beqa | 31.107 | | 34.851 |
| 14 (i) | *R. melanocephalus* | TAU.R16246 | KX909275 | KX909310 | KX909339 | KX909354 | Israel | Har Tayyasim | 31.771 | | 35.082 |
| 15 (i) * | *R. melanocephalus* | TAU.R16336 | KX909276 | KX909311 | - | KX909349 | Israel | Kefar Ha-Oranim | 31.920 | | 35.032 |
| 16 (i) * | *R. melanocephalus* | TAU.R15268 | KX909273 | KX909305 | KX909340 | KX909348 | Israel | Merav | 32.452 | | 35.423 |
| 17 (i) * | *R. melanocephalus* | TAU.R15269 | KT878841 | KT878845 | KT878853 | KT878849 | Israel | Merav | 32.452 | | 35.423 |
| 18 (i) * | *R. melanocephalus* | TAU.R16468 | KX909279 | KX909307 | KX909338 | KX909351 | Israel | Merav | 32.453 | | 35.422 |
| 19 (i) * | *R. melanocephalus* | TAU.R16575 | KX909278 | KX909308 | KX909341 | KX909352 | Israel | Shemurat Nahal Tanninim, Maagan Michael | 32.546 | | 34.915 |
| 20 (i) * | *R. melanocephalus* | TAU.R16622 | KX909274 | KX909309 | - | KX909355 | Israel | Nahal Oren, Karmel | 32.714 | | 34.985 |
| 21 (i) * | *R. melanocephalus* | TAU.R16340 | KX909277 | KX909306 | KX909342 | KX909350 | Israel | Montfort - upper Galilee | 33.047 | | 35.221 |
| 22 (i,ii) * | *R. melanocephalus* | HUJ.R20967 | KX909264 | KX909298 | - | KX909353 | Israel | Mt. Hermon | 33.299 | | 35.700 |
| 23 (i) * | *R. melanocephalus* | ZMHRU2007-69 | KT878840 | KT878844 | KT878852 | KT878848 | Syria | Tartus | 35.063 | | 35.899 |
| 24 (i) * | *R. melanocephalus* | ZDEU206/2006 | - | - | KR065433 | KR065425 | Turkey | Sofular Village, Harbiye, Hatay | 36.110 | | 36.173 |
| 25 (i) * | *R. melanocephalus* | ZDEU207/2006 | - | - | KR065434 | KR065426 | Turkey | Kuruyer Village, Harbiye, Hatay | 36.186 | | 36.194 |
| 26 (i) * | *R. satunini* | ZDEU125/2006 | - | - | KR065435 | KR065427 | Turkey | Nurdağı, Gaziantep | 37.171 | | 36.738 |
| 27 (i,ii) * | *R. satunini* | ZMHRU2015/0 | KX909263 | KX909297 | KX909328 | KX909362 | Turkey | Artuklu, Mardin | 37.335 | | 40.706 |
| 28 (i) * | *R. satunini* | ZDEU82/2007 | - | - | KR065436 | KR065428 | Turkey | Yalintepe, Cizre | 37.290 | | 42.058 |
| 29 (i,ii) * | *R. satunini* | CAS228723 | KT878843 | KT878846 | KT878855 | KT878850 | Iran | 40 km NW Kermanshah, 5 km W Lalabad Village (by air) | 34.584 | | 49.891 |

^1^ Specimen code abbreviations: [CAS] California Academy of Sciences, USA; [HUJ.R] National Natural History Collections, The Hebrew University of Jerusalem, Israel; [CN] Institute of Evolutionary Biology, Barcelona, Spain; [TAU.R] The Steinhardt Museum of Natural History, Israel National Centre for Biodiversity Studies, Tel Aviv University, Israel; [ZDEU] Zoology Department, Ege University, Turkey; [ZMHRU] Zoological Museum of Harran University, Osmanbey, Sanliurfa, Turkey.

(B)

| **Species** | **Specimen code^1^** | ***12S*** | ***16S*** | ***cytb*** | ***c-mos*** | **Country** | **Locality** | **Latitude** | **Longitude** |
| --- | --- | --- | --- | --- | --- | --- | --- | --- | --- |
| *Bamanophis dorri* |  | – | AY188081 | AY188040 | AY188001 |  |  |  |  |
| *Mopanveldophis zebrinus* |  | – | AY188084 | AY188043 | AY188004 |  |  |  |  |
| *Dolichophis caspius* |  | AY039135 | AY376768 | AY039173 | AY376797 |  |  |  |  |
| *Dolichophis jugularis* |  | AY039152 | AY376769 | AY486917 | AY486941 |  |  |  |  |
| *Eirenis aurolineatus* |  | – | AY376778 | AY376749 | AY376807 |  |  |  |  |
| *Eirenis barani* |  | – | AY376785 | AY376764 | AY376822 |  |  |  |  |
| *Eirenis collaris* |  | – | AY376795 | AY376766 | AY376824 |  |  |  |  |
| *Eirenis coronelloides* |  | – | AY376787 | AY376758 | AY376816 |  |  |  |  |
| *Eirenis decemlineatus* |  | – | AY376789 | AY376760 | AY376818 |  |  |  |  |
| *Eirenis eiselti* |  | – | AY376776 | AY376747 | AY376805 |  |  |  |  |
| *Eirenis levantinus* |  | – | AY376794 | AY376765 | AY376823 |  |  |  |  |
| *Eirenis lineomaculatus* |  | – | AY376791 | AY376762 | AY376820 |  |  |  |  |
| *Eirenis medus* |  | AY647226 | AY376796 | AY376767 | AY376825 |  |  |  |  |
| *Eirenis modestus* |  | AY039143 | AY376792 | AY486933 | AY486957 |  |  |  |  |
| *Eirenis punctatolineatus* |  | AY647227 | AY376781 | AY376755 | AY376813 |  |  |  |  |
| *Eirenis thospitis* |  | – | AY376790 | AY376761 | AY376819 |  |  |  |  |
| *Hemerophis socotrae* |  | AY039132 | AY188083 | AY188042 | AY188003 |  |  |  |  |
| *Hemorrhois algirus* |  | AY039149 | – | AY486911 | AY486935 |  |  |  |  |
| *Hemorrhois hippocrepis* |  | DQ451992 | – | DQ451987 | AY486940 |  |  |  |  |
| *Hemorrhois nummifer* |  | AY039163 | AY376771 | AY039201 | AY376800 |  |  |  |  |
| *Hemorrhois ravergieri* |  | AY039131 | – | AY486920 | AY486944 |  |  |  |  |
| *Hierophis gemonensis* |  | AY039145 | AY376770 | AY039183 | AY376799 |  |  |  |  |
| *Hierophis spinalis* |  | AY541508 | AY376773 | AY486924 | AY486948 |  |  |  |  |
| *Hierophis viridiflavus* |  | AY541505 | AY376774 | AY486925 | AY486949 |  |  |  |  |
| *Lytorhynchus diadema diadema* | IBECN4093 | KX909259 | KX909293 | KX909324 | KX909363 | Oman | 8 km N. of Al Kamil Wal Wafi | 22.308 | 59.221 |
| *Lytorhynchus diadema diadema* | SPM002589 | KX909261 | KX909295 | KX909326 | KX909364 | Egypt | North Sinai |  |  |
| *Lytorhynchus diadema diadema* | S1329 | KX909260 | KX909294 | KX909325 | KX909365 | Morocco | --- |  |  |
| *Lytorhynchus diadema gaddi* | MVZ234500 | KX909262 | KX909296 | KX909327 | – | Iran | sand dunes 53 km E Ahvaz via Ahvaz-Haftgel road | 49.236 | 31.273 |
| *Lytorhynchus maynardi* | MVZ234499 | KX909284 | KX909316 | KX909343 | KX909366 | Iran | sand dunes 6 km N (by air) Bampur | 27.253 | 60.409 |
| *Lytorhynchus maynardi* | MVZ248462 | KX909285 | KX909317 | KX909344 | KX909367 | Pakistan | Khalbat area , Zangi Nawar, 30 Km W Nushki, Chaghi district | 29.389 | 65.684 |
| *Lytorhynchus maynardi* | MVZ248463 | KX909286 | – | KX909345 | KX909368 | Pakistan | Khalbat area , Zangi Nawar, 30 Km W Nushki, Chaghi district | 29.389 | 65.684 |
| *Macroprotodon abubakeri* |  | AY643297 | AY643338 | AY643383 | – |  |  |  |  |
| *Macroprotodon brevis* |  | AY643291 | AY643332 | AY643374 | – |  |  |  |  |
| *Macroprotodon cucullatus* |  | AY643290 | AY188065 | AY188026 | AY187987 |  |  |  |  |
| *Muhtarophis barani* | ZMHRU2012-93 | KX909253 | KX909287 | KX909318 | KX909369 | Turkey | Amanos Mt, between Dörtyol and Hassa, Hatay | 36.8396 | 36.4186 |
| *Muhtarophis barani* | ZMHRU2014/60-1 | KX909254 | KX909288 | KX909319 | KX909370 | Turkey | Amanos Mt, between Dörtyol and Hassa, Hatay | 36.8401 | 36.4178 |
| *Muhtarophis barani* | ZMHRU2014/60-2 | KX909257 | KX909289 | KX909320 | KX909371 | Turkey | Amanos Mt, between Dörtyol and Hassa, Hatay | 36.8399 | 36.4175 |
| *Muhtarophis barani* | ZMHRU2014/60-3 | KX909258 | KX909290 | KX909321 | KX909372 | Turkey | Amanos Mt, between Dörtyol and Hassa, Hatay | 36.8395 | 36.4170 |
| *Muhtarophis barani* | ZMHRU2014/60-4 | KX909255 | KX909291 | KX909322 | KX909373 | Turkey | Amanos Mt, between Dörtyol and Hassa, Hatay | 36.8397 | 36.4176 |
| *Muhtarophis barani* | ZMHRU2014/60-5 | KX909256 | KX909292 | KX909323 | KX909374 | Turkey | Amanos Mt, between Dörtyol and Hassa, Hatay | 36.8397 | 36.4173 |
| *Oligodon arnensis* |  | KC347327 | KC347365 | KC347481 | KC347405 |  |  |  |  |
| *Oligodon cinereus* |  | HM591503 | HM591508 | AF471033 | AF471101 |  |  |  |  |
| *Oligodon sublineatus* |  | KC347329 | KC347367 | KC347483 | KC347407 |  |  |  |  |
| *Oligodon taeniolatus* |  | KC347330 | KC347368 | KC347484 | KC347408 |  |  |  |  |
| *Platyceps collaris* |  | AY039157 | – | AY486922 | AY486946 |  |  |  |  |
| *Platyceps florulentus* |  | AY039130 | – | AY486915 | AY486939 |  |  |  |  |
| *Platyceps karelini* |  | AY647232 | – | AY486918 | AY486942 |  |  |  |  |
| *Platyceps najadum* |  | AY039128 | – | AY486912 | AY486936 |  |  |  |  |
| *Platyceps rhodorachis* |  | AY039154 | – | AY486921 | AY486945 |  |  |  |  |
| *Platyceps rogersi* |  | AY039127 | AY188082 | AY188041 | AY188002 |  |  |  |  |
| *Spalerosophis diadema* |  | AY039144 | HQ658450 | AF471049 | AF471155 |  |  |  |  |
| *Wallaceophis gujaratensis* |  | KR819920 | – | – | KR819918 |  |  |  |  |
| *Wallaceophis gujaratensis* |  | KR819919 | KR819921 | – | KR819917 |  |  |  |  |

^1^ Specimen code abbreviations: [IBE, S, SPM] Institute of Evolutionary Biology, Barcelona, Spain; [MVZ] Museum of Vertebrate Zoology, University of California, Berkeley, USA; [ZMHRU] Zoological Museum of Harran University, Osmanbey, Sanliurfa, Turkey.

(C)

|  | ***R. melanocephalus*** | ***R. arabicus*** | ***R. dayanae* sp. nov.** | ***R. satunini*** |
| --- | --- | --- | --- | --- |
| ***R. melanocephalus*** | 0.6/0.2/1 | 10.5 | 10.2 | 13 |
| ***R. arabicus*** | 7.3/3.7 | -/-/- | 10 | 12.8 |
| ***R. dayanae* sp. nov.** | 6.7/4 | 5.9/4.2 | 0.1/0.3/0.5 | 11.9 |
| ***R. satunini*** | 9.1/3.9 | 8.8/4.5 | 7.3/5.4 | 1/0.4/1.8 |
