# Supplementary material for: An integrative systematic revision and biogeography of Rhynchocalamus snakes (Reptilia, Colubridae) with a description of a new species from Israel
Source: PeerJ. 2016 Dec 22;4:e2769. doi: 10.7717/peerj.2769 (PMC5183090; doi:10.7717/peerj.2769)
Supplement: Table S2 — (A) Species-set A; (B) species-set B; (C) species-set C. [file peerj-04-2769-s002.docx]

(A) Species-set A - the Western Palearctic Colubrinae clade

| **Analysis** | **Dataset** | **Partition** | **Length** | **Model** | **Clock model (unlinked)** | **Rate** | **Tree model (linked)** | **Other Priors/Parameters** | | **Runs** | | |
| --- | --- | --- | --- | --- | --- | --- | --- | --- | --- | --- | --- | --- |
| Phylogenetic  trees  **Maximum**  **Likelihood** | mtDNA+nDNA  (N=82)  **Dataset 1** | *12S* | 581 | GTR+G |  | | | | Replicates x100  Bootstrap x1000 | |  |  |
|  |  | *16S* | 505 |  |  |  |  |  |  |  |  |  |
|  |  | *cytb* codons 1+2 | 664 |  |  |  |  |  |  |  |  |  |
|  |  | *cytb* codon 3 | 332 |  |  |  |  |  |  |  |  |  |
|  |  | c-*mos* codon 1+2 | 272 |  |  |  |  |  |  |  |  |  |
|  |  | c-*mos* codon 3 | 136 |  |  |  |  |  |  |  |  |  |
| Phylogenetic  trees  **Bayesian**  **Inference** |  | *12S* | 581 | GTR+I+G | Relaxed Uncorrelated Lognormal | Rate fixed to 1 | Yule  Random starting tree | Alpha Uniform (0, 10); Base substitution Uniform (0, 100) | | 3 runs;  10^8^ generations;  10^4^ sampling frequency;  10% burn-in | |  |
|  |  | *16S* | 505 | GTR+I+G | Relaxed Uncorrelated Lognormal |  |  |  |  |  |  |  |
|  |  | *cytb* codons 1+2 | 664 | GTR+I+G | Relaxed Uncorrelated Lognormal |  |  |  |  |  |  |  |
|  |  | *cytb* codon 3 | 332 | GTR+G | Relaxed Uncorrelated Lognormal |  |  |  |  |  |  |  |
|  |  | c-*mos* codons 1+2 | 272 | TrN+I+G | Strict clock |  |  |  |  |  |  |  |
|  |  | c-*mos* codon 3 | 136 | TrN+G | Strict clock |  |  |  |  |  |  |  |
| Estimation of divergence times  **Bayesian**  **Inference** | mtDNA+nDNA  (N=48)  **Dataset 2**  Including one representative of each mPTP entity for *Rhynchocalamus* only | *12S* | 583 | GTR+I+G | Relaxed Uncorrelated Lognormal | 0.0001 (0-1) | Yule  Random starting tree | Alpha Uniform (0, 10); Base substitution Uniform (0, 100) | | 3 runs;  3x10^8^ generations;  3x10^4^ sampling frequency;  10% burn-in | |  |
|  |  | *16S* | 507 | GTR+I+G | Relaxed Uncorrelated Lognormal |  |  |  |  |  |  |  |
|  |  | *cytb* codons 1+2 | 664 | GTR+I+G | Relaxed Uncorrelated Lognormal |  |  |  |  |  |  |  |
|  |  | *cytb* codon 3 | 332 | GTR+G | Relaxed Uncorrelated Lognormal |  |  |  |  |  |  |  |
|  |  | c-*mos* codons 1+2 | 272 | TrN+I | Strict clock |  |  |  |  |  |  |  |
|  |  | c-*mos* codon 3 | 136 | GTR+G | Strict clock |  |  |  |  |  |  |  |

(B) Species-set B - *Rhynchocalamus* and *Lytorhynchus*

| **Analysis** | **Dataset** | **Partition** | **Length** | **Model** | **Clock model (unlinked)** | **Rate** | **Tree model (linked)** | **Other Priors/Parameters** | | **Runs** |
| --- | --- | --- | --- | --- | --- | --- | --- | --- | --- | --- |
| Phylogenetic  trees  **Maximum**  **Likelihood** | mtDNA+nDNA  (N=36)  **Dataset 3** | *12S* | 618 |  | | | | | Replicates x100  Bootstrap x1000 | |
|  |  | *16S* | 510 |  |  |  |  |  |  |  |
|  |  | *cytb* codons 1+2 | 728 |  |  |  |  |  |  |  |
|  |  | *cytb* codon 3 | 364 |  |  |  |  |  |  |  |
|  |  | c-*mos* codon 1+2 | 272 |  |  |  |  |  |  |  |
|  |  | c-*mos* codon 3 | 136 |  |  |  |  |  |  |  |
| Phylogenetic  trees  **Bayesian**  **Inference** |  | *12S* | 618 | GTR+G | Relaxed Uncorrelated Lognormal | Rate fixed to 1 | Yule  Random starting tree | Alpha Uniform (0, 10); Base substitution Uniform (0, 100) | | 3 runs;  8x10^7^ generations;  8x10^3^ sampling frequency;  10% burn-in |
|  |  | *16S* | 510 | GTR+I+G | Relaxed Uncorrelated Lognormal |  |  |  |  |  |
|  |  | *cytb* codons 1+2 | 728 | HKY+I | Relaxed Uncorrelated Lognormal |  |  |  |  |  |
|  |  | *cytb* codon 3 | 364 | GTR+G | Relaxed Uncorrelated Lognormal |  |  |  |  |  |
|  |  | c-*mos* codons 1+2 | 272 | HKY | Strict clock |  |  |  |  |  |
|  |  | c-*mos* codon 3 | 136 | HKY | Strict clock |  |  |  |  |  |

(C) Species-set C - *Rhynchocalamus* only

| **Analysis** | **Dataset** | **Partition** | **Length** | **Model** | **Other Priors/Parameters** |
| --- | --- | --- | --- | --- | --- |
| Phylogenetic  trees  **Maximum**  **Likelihood** | mtDNA  Haplotypes (N=28)  **Dataset 4** | *12S* | 618 |  |  |
|  |  | *16S* | 510 | GTR+G | Replicates x100  Bootstrap x1000 |
|  |  | *cytb* codons 1+2 | 728 |  |  |
|  |  | *cytb* codon 3 | 364 |  |  |
