# Supplementary material for: An integrative systematic revision and biogeography of Rhynchocalamus snakes (Reptilia, Colubridae) with a description of a new species from Israel
Source: PeerJ. 2016 Dec 22;4:e2769. doi: 10.7717/peerj.2769 (PMC5183090; doi:10.7717/peerj.2769)
Supplement: Table S4 — P-values in bold indicate high level of statistical significance. Abbreviations of each morphological variable, as in Table S3, are listed in the Material and Methods section. [file peerj-04-2769-s004.docx]

A) Mensural characters (one-way ANCOVA; SVL as a covariate for size correction; Adult specimens only; *permutation test)

Sexual dimorphism - *R. melanocephalus*

|  | **TL*** | **PilL*** | **RL** | **RH** | **RW** | **FL** | **FW** | **PL** | **PW** | **IntNL*** | **PFL** | **EYE** | **BPL** | **InD*** | **AimL** | **PimL** |
| --- | --- | --- | --- | --- | --- | --- | --- | --- | --- | --- | --- | --- | --- | --- | --- | --- |
| D.f. | 92 | 91 | 89 | 81 | 89 | 91 | 91 | 87 | 90 | 86 | 87 | 91 | 88 | 89 | 79 | 77 |
| F | --- | --- | 11.566 | 5.905 | 16.858 | 10.528 | 14.215 | 10.409 | 9.227 | --- | 1.905 | 13.125 | 9.891 | --- | 18.073 | 12.748 |
| Pr >F | 0.173 | **0.002** | **0.001** | **0.017** | **<0.0001** | **0.002** | **<0.0001** | **0.002** | **0.003** | 0.784 | 0.171 | **<0.0001** | **0.002** | **0.001** | **<0.0001** | **0.001** |

Species differences - *R. melanocephalus vs. R. dayanae* sp. nov.

|  | **TL*** | **PilL** | **RL** | **RH** | **RW** | **FL** | **FW** | **PL** | **PW** | **IntNL** | **PFL** | **EYE** | **BPL** | **InD** | **AimL** | **PimL** |
| --- | --- | --- | --- | --- | --- | --- | --- | --- | --- | --- | --- | --- | --- | --- | --- | --- |
| D.f. | 61 | 59 | 58 | 50 | 57 | 59 | 59 | 57 | 57 | 56 | 57 | 48 | 58 | 57 | 48 | 46 |
| F | --- | 0.445 | 0.054 | 9.885 | 2.323 | 1.658 | 0.002 | 0.324 | 4.651 | 0.994 | 3.456 | 0.048 | 12.440 | 18.344 | 2.922 | 0.403 |
| Pr >F | 0.706 | 0.507 | 0.817 | **0.003** | 0.133 | 0.203 | 0.966 | 0.572 | **0.035** | 0.323 | 0.068 | 0.827 | **0.001** | **<0.0001** | 0.094 | 0.525 |

Species differences - *R. melanocephalus* (southern population) *vs. R. dayanae* sp. nov.

|  | **TL** | **PilL** | **RL** | **RH** | **RW** | **FL** | **FW** | **PL** | **PW** | **IntNL** | **PFL** | **EYE** | **BPL** | **InD** | **AimL** | **PimL** |
| --- | --- | --- | --- | --- | --- | --- | --- | --- | --- | --- | --- | --- | --- | --- | --- | --- |
| D.f. | 9 | 9 | 9 | 8 | 8 | 9 | 9 | 9 | 8 | 9 | 9 | 8 | 9 | 7 | 8 | 8 |
| F | 2.988 | 0.693 | 1.284 | 8.714 | 0.280 | 1.878 | 0.003 | 3.204 | 17.414 | 1.672 | --- | 3.035 | 1.189 | 23.974 | 4.320 | 4.716 |
| Pr >F | 0.122 | 0.429 | 0.286 | **0.021** | 0.613 | 0.208 | 0.955 | 0.111 | **0.004** | 0.232 | 0.088 | 0.125 | 0.307 | **0.003** | 0.076 | 0.066 |

B) SVL, meristic characters (one-way ANOVA; * permutation test)

Sexual dimorphism - *R. melanocephalus*

|  | **SVL*** | **VS*** | **SCS*** | **BDS*** | **DST*** |
| --- | --- | --- | --- | --- | --- |
| D.f. | 93 | 91 | 98 | 94 | 99 |
| F | --- | --- | --- | --- | --- |
| Pr >F | 0.06 | 0.076 | 0.08 | 0.295 | 0.192 |

Species differences - *R. melanocephalus vs. R. dayanae* sp. nov.

|  | **SVL*** | **VS*** | **SCS*** | **BDS*** | **DST*** |
| --- | --- | --- | --- | --- | --- |
| D.f. | 61 | 62 | 66 | 63 | 67 |
| F | --- | **---** | --- | **---** | --- |
| Pr >F | 0.42 | **0.015** | 1 | **<0.0001** | 1 |

Species differences - *R. melanocephalus* (southern population) *vs. R. dayanae* sp. nov.

|  | **SVL** | **VS** | **SCS** | **BDS** | **DST** |
| --- | --- | --- | --- | --- | --- |
| D.f. | 10 | 9 | 11 | 11 | 11 |
| F | 0.626 | 5.939 | 0.133 | 0.027 | 3.386 |
| Pr >F | 0.449 | **0.041** | 0.723 | 0.872 | 0.096 |

C) Meristic characters (Fisher’s exact probability test)

Sexual dimorphism - *R. melanocephalus*

|  | **InfLC** | **LL** | **PostO** |
| --- | --- | --- | --- |
| Pr >F | 0.731 | 0.217 | 0.179 |

Species differences - *R. melanocephalus vs. R. dayanae* sp. nov.

|  | **InfLC** | **LL** | **PostO** |
| --- | --- | --- | --- |
| Pr >F | **<0.0001** | **<0.0001** | **<0.0001** |

Species differences - *R. melanocephalus* (southern population) *vs. R. dayanae* sp. nov.

|  | **InfLC** | **LL** | **PostO** |
| --- | --- | --- | --- |
| Pr >F | **<0.0001** | **<0.0001** | **0.006** |
