# Supplementary figures and images for: An integrative systematic revision and biogeography of Rhynchocalamus snakes (Reptilia, Colubridae) with a description of a new species from Israel
Source: PeerJ. 2016 Dec 22;4:e2769. doi: 10.7717/peerj.2769 (PMC5183090; doi:10.7717/peerj.2769)

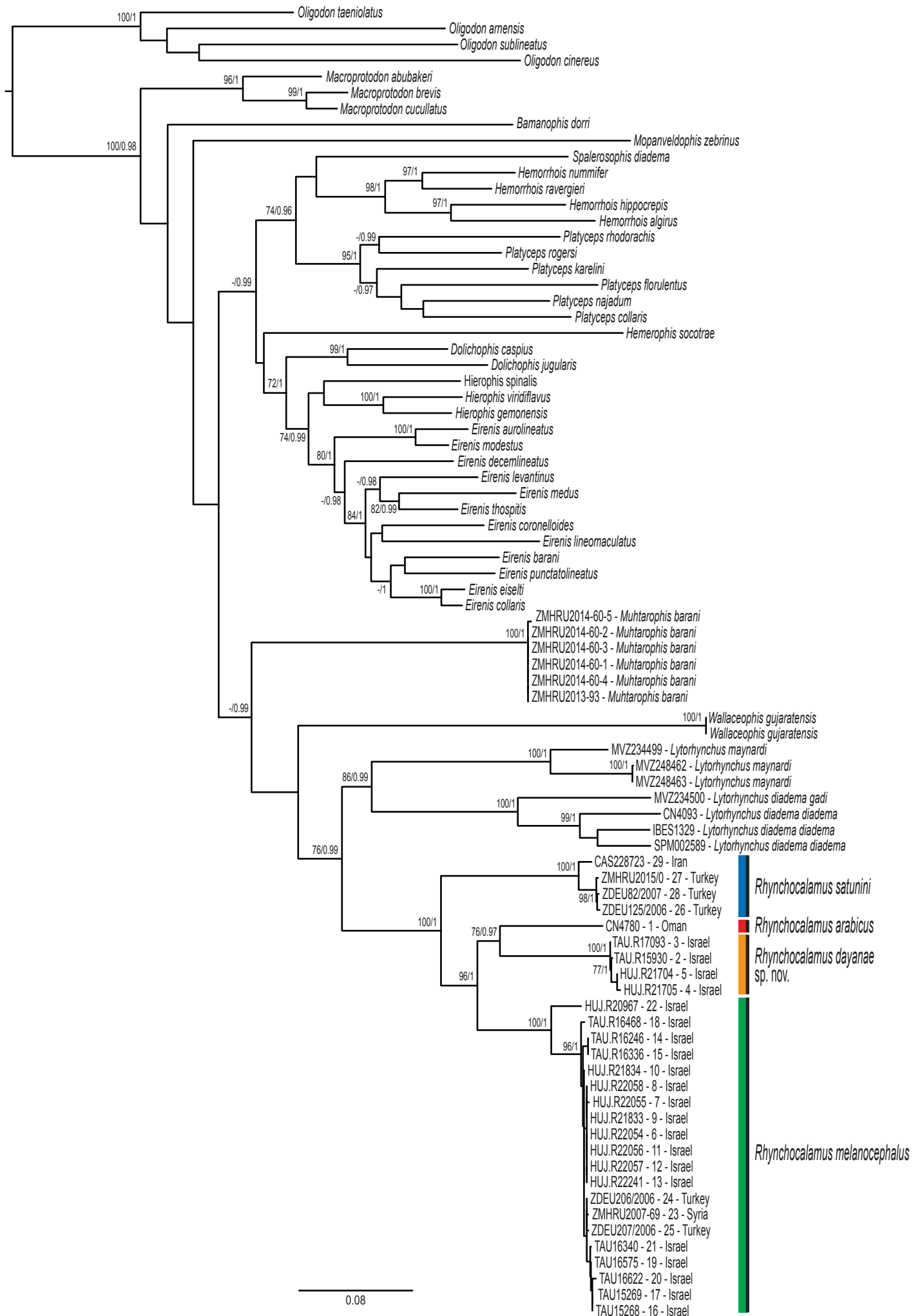

Supplement: Figure S1 — The tree was inferred from the concatenated dataset of the mitochondrial (12S, 16S, cytb) and nuclear (c-mos) gene fragments (dataset 1). Support values near the nodes indicate ML bootstrap and BI posterior probability (values ≥ 70%, ≥ 0.95; ML, BI, respectively). Sample codes and colours correlate to specimens in Table S1 and in Figs. 1–2,Tables S2–S4. Taxon names correspond to changes proposed in this paper. [file peerj-04-2769-s005.pdf]

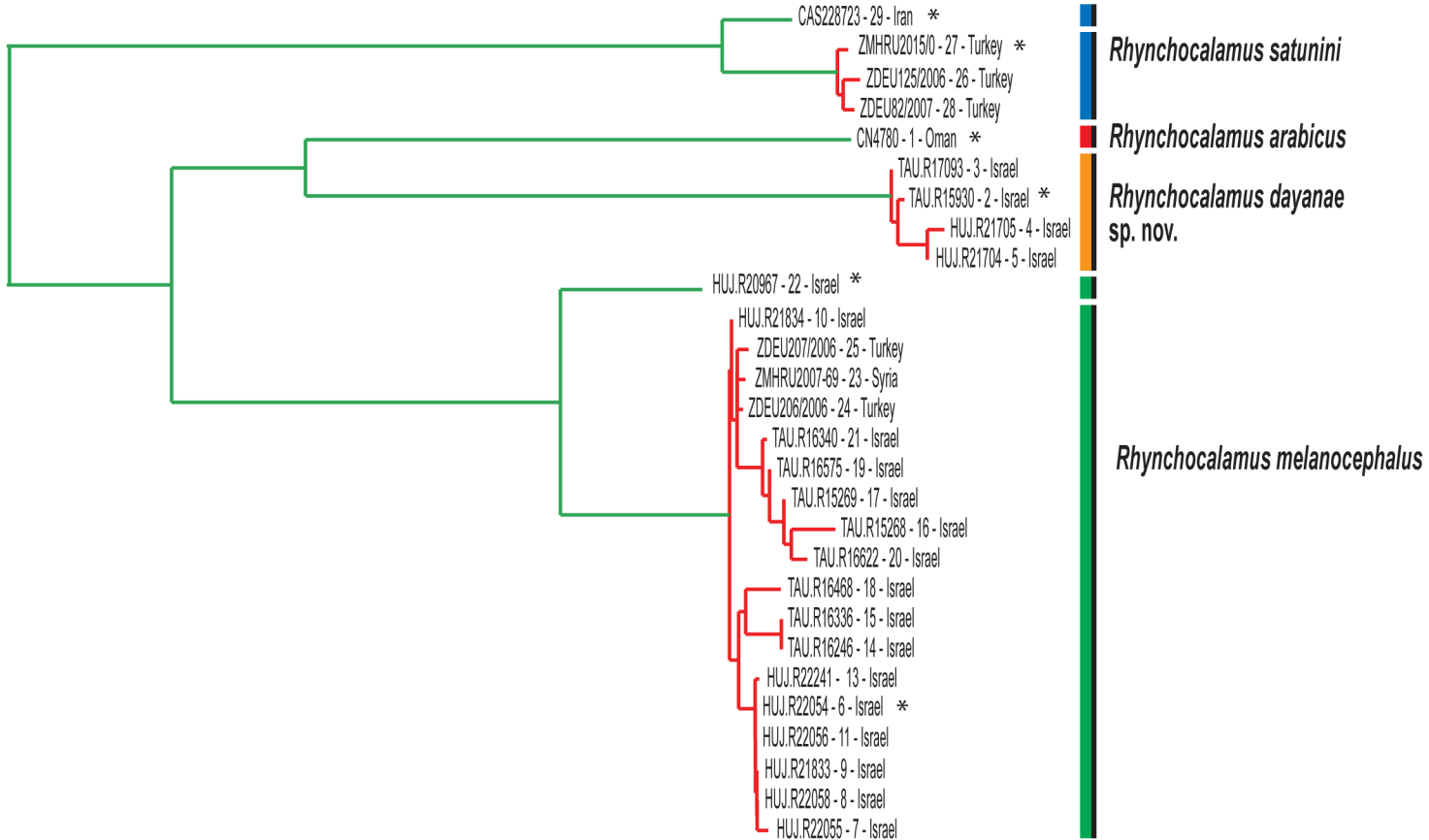

Supplement: Figure S2 — The Analysis was based on haplotype mtDNA (dataset 4). Asterisk indicates representatives used for the divergence time estimation analysis. Sample codes correlate to specimens in Table S1 and in Figs. 1–2,Tables S1–S3. [file peerj-04-2769-s006.pdf]

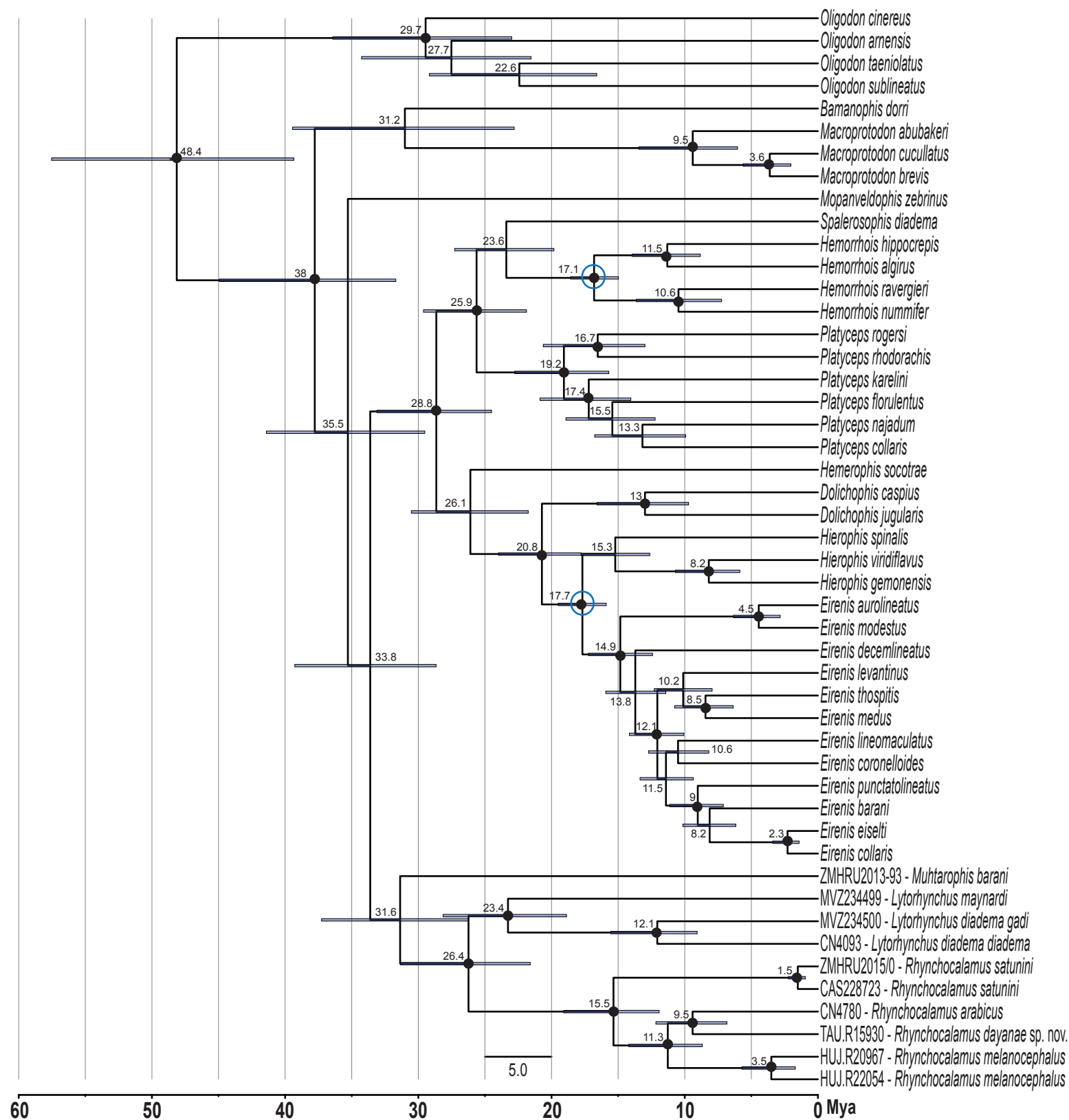

Supplement: Figure S3 — The tree was inferred from the concatenated dataset of the mitochondrial (12S, 16S, cytb) and nuclear (c-mos) gene fragments (dataset 1), based on two calibration points of Hemorrhois and Hierophis subgroups (blue circles; see Material and Methods; dataset 2). Median age estimates are provided near the nodes with bars representing the 95% highest posterior densities (HPD). Black circles represent nodes with high support (posterior probability values ≥ 0.95). Sample codes correlate to specimens in Table S1 and in Figs. 1–2,Tables S1–S2. [file peerj-04-2769-s007.pdf]

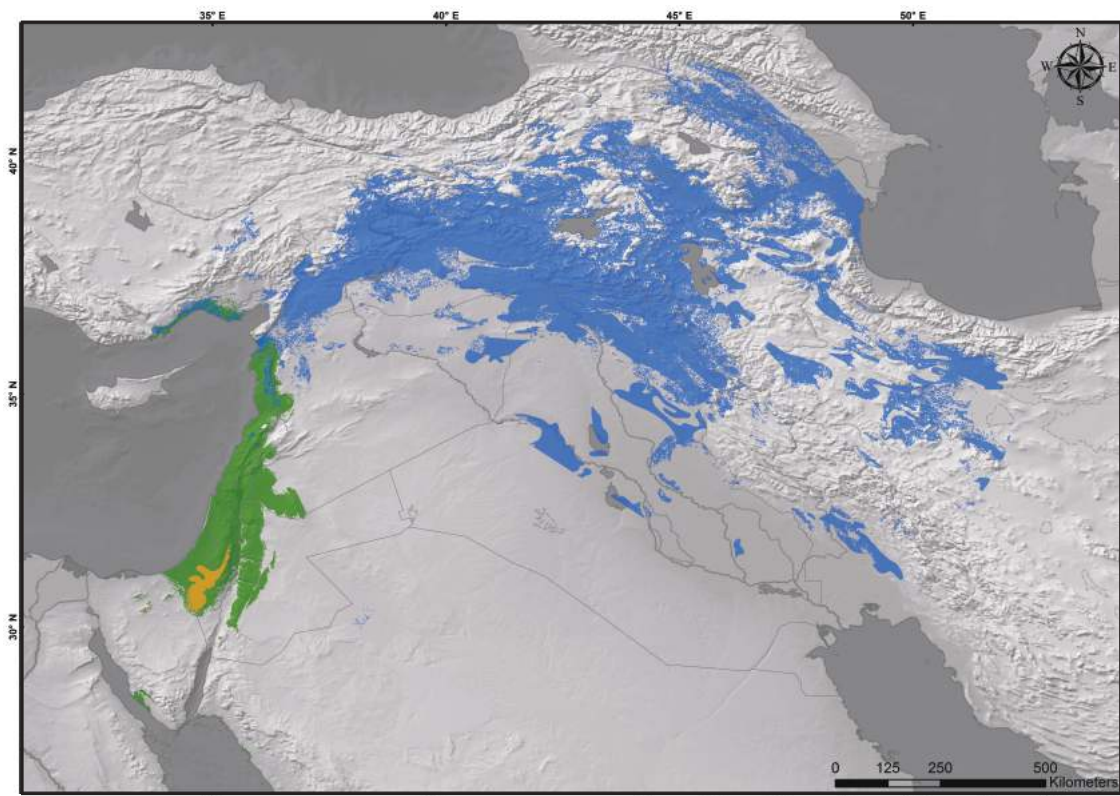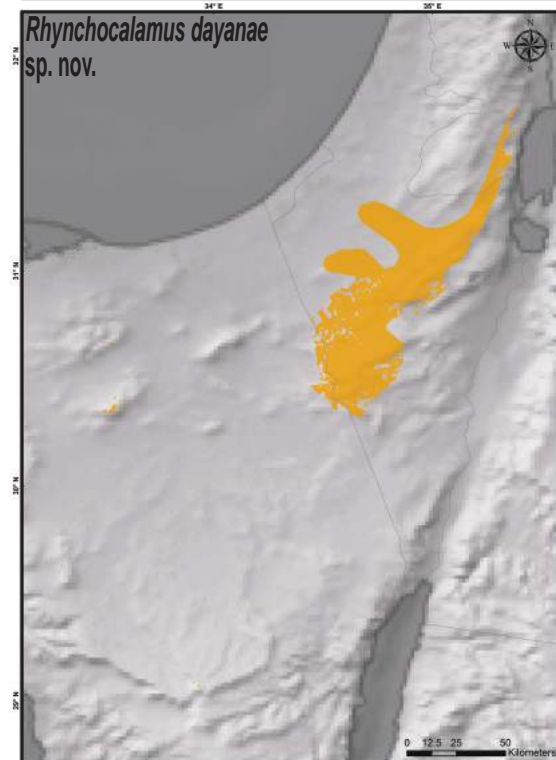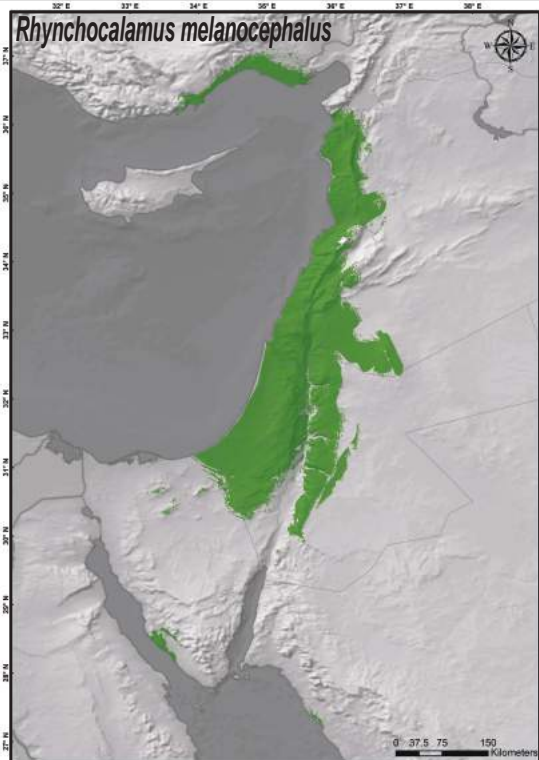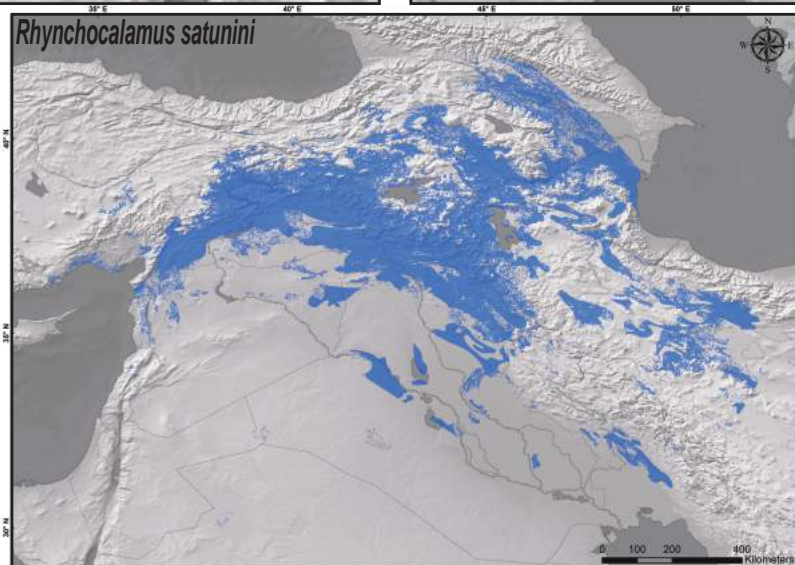

Supplement: Figure S4 — Projected Maxent models of the potential presence/absence of R. dayanae sp. nov., R. melanocephalus and R. satunini. The cut-off threshold was Maximum Training Sensitivity plus Specificity (MTSS) to convert the continuous Maxent output to binary maps. Colours correspond to taxa in Table S1 and in Figs. 1–2, Figs. S1–S2. [file peerj-04-2769-s008.pdf]
